# Macrogenetic Alignment in Ecological Strategies Better Interprets Assembly Processes Than Pre‐Determined Functional Groupings

**DOI:** 10.1111/ele.70418

**Published:** 2026-06-07

**Authors:** Maurizio Rossetto, Richard Dimon, Robert M. Kooyman, Peter D. Wilson, Jia‐Yee S. Yap

**Affiliations:** ^1^ Research Centre for Ecosystem Resilience Botanic Gardens of Sydney Sydney New South Wales Australia; ^2^ Queensland Alliance of Agriculture and Food Innovation University of Queensland St Lucia Queensland Australia; ^3^ School of Natural Sciences Macquarie University Sydney New South Wales Australia

**Keywords:** dominant trees, ecosystem types, genomic patterns, landscape processes, plant assemblages, population genetics, rainforests, species groups

## Abstract

Understanding how species assemble across landscapes requires integration of data representing evolutionary, ecological, and biogeographic processes. We developed a comparative macrogenetic framework, applying it across 22 co‐distributed rainforest trees, to identify replicated landscape‐level genetic signatures. Diversity‐migration analyses and genogeographic clustering identified shared spatial dynamics in relation to refugial areas and genetic turnover, but with no direct relation to simple functional trait combinations. Three broad patterns emerged: *Higher Northern Diversity* with southward migration, *Higher Southern Diversity* with northward migration, and *Homogeneous Diversity* with no directional migration. We identified five (post hoc) species groups sharing gene flow and isolation‐by‐distance dynamics in relation to recognised biogeographic barriers. Replicated genetic signatures highlight how assembly processes emerge from interacting ecological and historical filters rather than single traits or biogeographic histories alone. We present a statistically replicable interpretational framework to identify shared evolutionary and ecological dynamics, offering scalable, management relevant tools to support restoration planning and biodiversity conservation under environmental change across all types of vegetation.

## Graphical abstract

A comparative macrogenetic framework reveals that standardised landscape‐genomics can identify shared patterns reflecting common histories and assembly processes. We detected convergence in distributional dynamics and delineate three broad patterns and five species groups defined by gene flow and isolation‐by‐distance, highlighting the limitations of trait‐based generalisations and supporting more robust, evidence‐based management strategies.

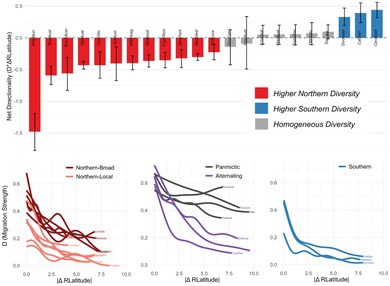

## 1 Introduction

The establishment and persistence of plant assemblages is regulated by factors that filter the local pool of species according to their environmental tolerances, trait combinations and relative competitive advantages (Kunstler et al. 2016; Vellend 2016). The identification of the factors that regulate species distribution and co‐occurrence across spatial and temporal scales can guide increasingly sophisticated biodiversity management strategies (Brudvig 2017), including in the context of pervasive anthropogenic disturbances and the ensuing extinction escalation (Forzieri et al. 2022; Turvey and Crees 2019).

Coexistence (Chesson 2000, 2018) is shaped by differences in population dynamics and successional strategies reflecting species' relative abundance and persistence times in assemblages (Hubbell 2001; C. O. Webb 2000) and by the interaction of individual functional characteristics (Falster et al. 2017) with present‐day environmental conditions (Munoz et al. 2023). Those factors are, in turn, set within the constraints of the historical legacies that regulated turnover of individuals and genes (Barabás et al. 2018). Consequently, understanding vegetation assembly processes involves components of evolutionary biology and biogeography, as well as population and community ecology (HilleRisLambers et al. 2012; Kooyman et al. 2011).

Multiple studies have shown that highly biodiverse forested systems generally comprise large numbers of rare tree species (the long‐tailed distribution) and a small percentage of hyper dominants (Hubbell 2001; Slik et al. 2015; Ter Steege et al. 2020). The practical implications of this perspective are that categorising representative groupings of common, foundational species should be an effective way to investigate the broader factors shaping assembly‐level processes, predicting direction(s) of compositional change and supporting biodiversity management strategies. However, despite the identification of consistent global patterns in the distribution of commonness (as a proportion of dominant taxa), these patterns do not provide easily identifiable causal mechanisms (Cooper et al. 2024).

As an individual's genome determines its adaptability and the expression of the species fundamental niche, the landscape‐level partitioning and structuring of genetic diversity across a species' distribution has a role in regulating local competitive interactions (Ehlers et al. 2016). Consequently, comparative phylogeographic studies have been used to identify local landscape features that can impose temporal changes in population connectivity and dynamics (Avise 2000; Hickerson et al. 2007; Edwards et al. 2022). Within this context, species with analogous genetic signatures of diversity and temporal connectivity across the landscape can provide valuable insights into shared filtering processes that, through time, have led to contemporary assemblages (Ornelas et al. 2013; Exposito‐Alonso et al. 2022; Riginos and Jahnke 2023).

The search for universal evolutionary interpretations about assembly processes that could broadly guide biodiversity management has led to dedicated meta‐analyses exploring associative patterns between functional attributes and a range of simple genetic indices (e.g., global flora: Hamrick and Godt 1996; Australian flora: Broadhurst et al. 2017; rainforest flora: Kooyman et al. 2011). While such mechanistic approaches predominantly aim to link predefined traits to a species' competitive success or vulnerability, ultimately their interpretative power is constrained by data incompatibilities across studies reflecting sampling strategies, analytical methods and genetic measures. More importantly, these studies are limited by a reliance on set expectations in relation to the detection of associative patterns between genetic metrics and pre‐determined functional or distributional attributes.

The advent of new genetic and genomic methodologies that enable the gathering of multispecies datasets cost‐effectively (Supple and Shapiro 2018), and the emergence of standardised workflows for sampling and analysing them (Rossetto et al. 2019), promise a coherent and unified opportunity to overcome previous limitations. Comparative and standardised macrogenetic studies (Leigh et al. 2021) have the potential to remove data incompatibilities and explicitly target interactions between geographic and functional processes (Epps and Keyghobadi 2015; McGaughran et al. 2022). Consequently, it should be possible to identify groups of taxa displaying similar landscape‐level genetic signatures and subsequently document group‐specific combinations of distinguishing functional, biogeographic and environmental attributes. By primarily interrogating landscape dynamics (evolutionary processes) to define groupings rather than focusing on simple comparative phylogeography, researchers could circumvent the interpretative constraints associated with using predetermined functional (or other) factors to explain assembly processes. An approach that quantifies similarities among landscape‐level genetic signatures within a statistically validated framework, specifically to retrospectively identify the emerging characteristics interacting with assembly‐level processes, is yet to be formulated or tested.

Put simply, our working hypothesis sought to test the idea that: if emergent distribution‐wide genetic groupings could be detected they would have greater interpretative power than groupings based on a set of ‘a priori’ identified functional, ecological, phylogenetic or environmental factors. Consequently, we asked the following questions: (1) can we develop an innovative approach to explore assembly level processes based on statistically determined emergent species‐groupings that are independent of prior‐based expectations, and (2) more specifically, can we develop a replicable, scalable, macrogenetic approach comparing co‐distributed, widespread species to provide a novel interpretational framework to understand assembly‐level processes and potentially guide the development of vegetation management strategies? To address these questions, we employed a standardised landscape genetics sampling workflow developed by Rossetto et al. (2019) on 22 broadly co‐distributed Australian sub‐tropical rainforest trees to identify taxa displaying similar landscape‐level genetic signatures. Here we statistically evaluate and document emergent genetic groupings, and retrospectively identify ecologically informative combinations of distinguishing functional, biogeographic and environmental attributes. We rely on a multistep approach that integrates analyses of spatial genetic diversity, migration directionality, and migration strength, with genogeographic clustering to identify statistically supported species groups that can be placed into broad explorative ecological and conservation contexts.

## 2 Materials and Methods

### 2.1 Study System and Sampling Strategy

We selected the subtropical rainforests of eastern Australia as a study system because it has a well‐documented evolutionary and biogeographic history that allows for robust interpretation of genetic patterns. The continental distribution of the Australian rainforest vegetation of Gondwanan origins started to contract as the Sahul plate moved north starting around 40 Mya (Greenwood and Christophel 2005), becoming increasingly localised to the east and northeast of the continent as drier conditions intensified in the last 20 Mya (Miocene; Kooyman et al. 2014). Intensifying habitat disturbances and the increasing proximity to the Sunda shelf facilitated waves of continental invasions of rainforest lineages from Southeast Asia, particularly in more northerly and less stable areas (Crayn et al. 2015; Joyce et al. 2021; Yap et al. 2018). More recently, as the Quaternary ushered in a new era of cyclical environmental extremes, the cool dry conditions of glacial peaks caused rainforest contractions into refugia, followed by interglacial expansions into newly available habitat (Kooyman et al. 2013; Worth et al. 2017). Increased habitat availability during the interglacial periods, aligned with rainforest expansion‐contraction dynamics, also facilitated expansions from refugia into broader distributional ranges (Das et al. 2019; Yap et al. 2020).

**FIGURE 1:**
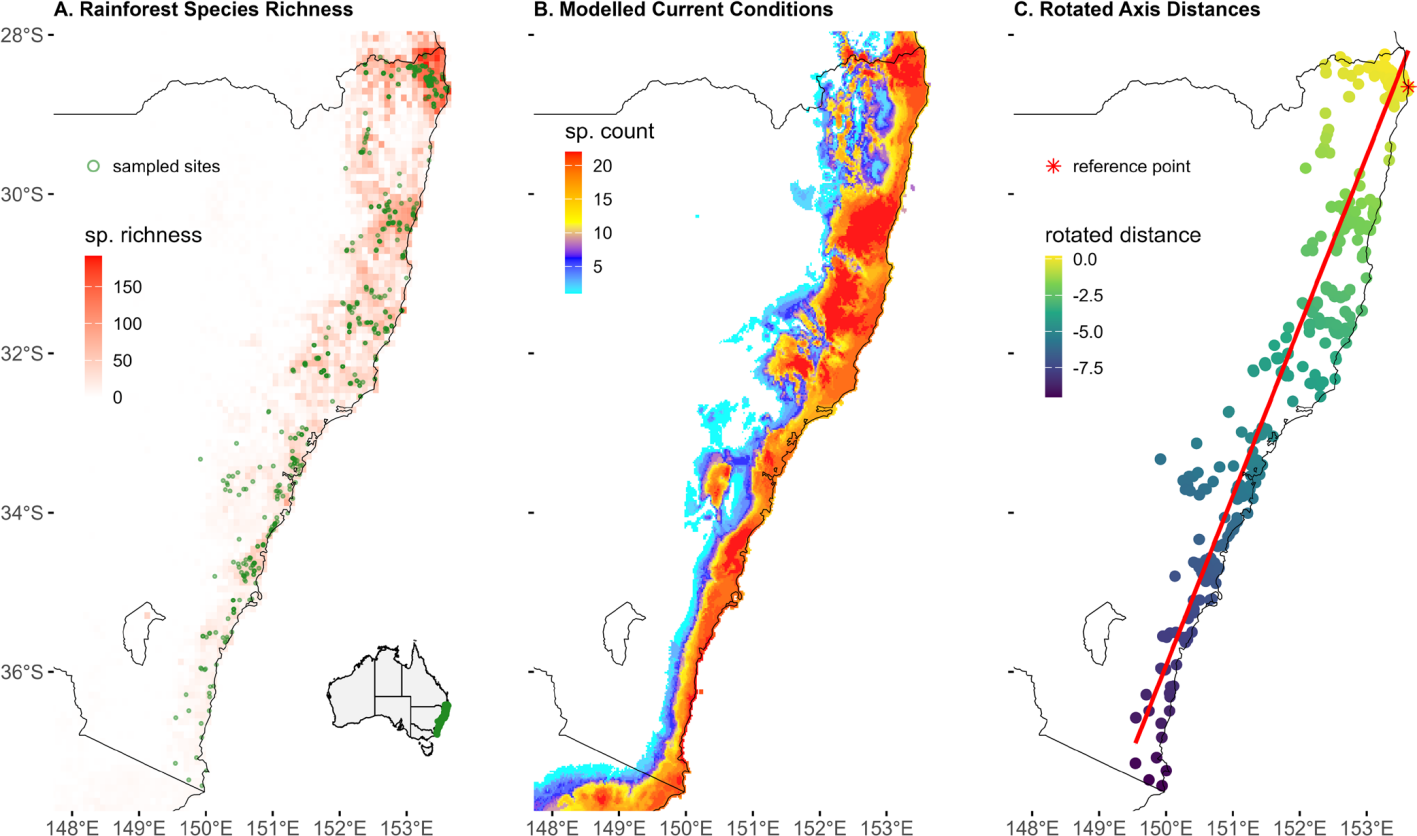
Overview of rainforest sampling along the east coast of New South Wales (NSW, Australia). (A) Location of genetic sampling (green circles) of the 22 study species overlaid with woody rainforest species richness (sp. richness; 50 × 50 km grid cells ranging from 0 to 192 species) from Kooyman et al. (2013) in the background. (B) Modelled distributional overlap under current conditions for the 22 target species with the number of overlapping species (sp. count) represented by the colour of raster cells. (C) Illustration of the spatial linearisation procedure, showing the fitted linear trend through all sampling sites (red line) and the associated rotation matrix *R* applied to site coordinates (each site represented by a dot). Dot colour represents distance along the rotated geographic axis, measured relative to a fixed reference point (*), defined as the northernmost predicted point along the fitted regression line.

We selected 22 rainforest tree species with broad phylogenetic, functional and biogeographic representation (20 genera and 15 families; Table 1), chosen for their relatively common, broadly overlapping distributions across eastern New South Wales (NSW, Australia). The geopolitical boundary of NSW provides an ideal case study encompassing the southern limit of warm temperate rainforests to the south, and the McPherson Range/Brisbane Valley Barrier (Bryant and Krosch 2016) near the Queensland border to the north. Sampling followed the standardised Restore & Renew workflow (Rossetto et al. 2019), targeting all core rainforest habitats across each species' extant NSW range (Figure 1A). Leaf material was collected from 5 to 6 individuals per site, spaced approximately ≥ 10 m apart. Sites were defined as ≥ 4 successfully sequenced individuals and separated by ≥ 3 km, with priority given to locations where species co‐occurred (Figure 1A,B). We generated sequence data for 3345 samples from 671 sampling locations across 22 rainforest tree species, with some sites overlapping among species where they co‐occurred, covering their distributions across New South Wales (Table S1.1).

**TABLE 1:** Study species (representing 20 genera from 15 families) grouped according to the genetic findings of this study and described by relevant biogeographic, ecological and functional characters including: Family; biogeographic origins (Origins); rainforest ecosystem types: CNVF—complex notophyll vine forest (complex sub‐tropical), NVF—notophyll vine forest (either littoral or simplified CNVF), ANVF—araucarian notophyll vine forest (seasonally drier vine forest), SNVF—simple notophyll vine forest, MFF—microphyll fern forest (L. Webb 1968); growth form; maximum height (m); wood density; dispersal type; seed size in mm (length × width).

Group	Species	Family	Origins	Ecosystem type	Growth form	Max height (m)	Wood density	Dispersal type	Seed size (mm)
*Panmictic*	*Ficus coronata*	Moraceae	Sunda	CNVF—NVF	Small tree	20	399.9	Frugivore	1 × 1
	*Pittosporum undulatum*	Pittosporaceae	Sunda	Rainforest margins	Small tree	20	731	Frugivore	3 × 1
	*Planchonella australis*	Sapotaceae	Sunda	CNVF	Canopy tree	40	756.8	Frugivore	20 × 10
	*Toona ciliata*	Meliaceae	Sunda	CNVF	Canopy persistent secondary	45	365.5	Wind	5 × 2
*Alternating*	*Cryptocarya glaucescens*	Lauraceae	Sahul	SNVF—NVF	Canopy tree	45	541.8	Frugivore	10 × 15
	*Schizomeria ovata*	Cunoniaceae	Sahul	SNVF—NVF	Canopy tree	35	567.6	Frugivore	11 × 8
	*Tristaniopsis laurina*	Myrtaceae	Sahul	SNVF—NVF riparian	Canopy tree	30	825.6	Wind	5 × 6
*Southern*	*Callicoma serratifolia*	Cunoniaceae	Sahul	SNVF	Canopy persistent secondary	35	494.5	Wind	1 × 1
	*Ceratopetalum apetalum*	Cunoniaceae	Sahul	SNVF—MFF	Canopy tree	40	537.5	Wind	2 × 1
	*Doryphora sassafras*	Atherospermataceae	Sahul	Widespread	Canopy tree	45	516	Wind	2 × 2
*Northern‐Broad*	*Cinnamomum oliveri*	Lauraceae	Sunda	SNVF‐ CNVF	Canopy tree	25	481.6	Frugivore	14 × 10
	*Diospyros pentamera*	Ebenaceae	Sahul	CNVF	Canopy tree	25	627.8	Frugivore	10 × 3
	*Orites e×celsus*	Proteaceae	Sahul	CNVF—SNVF	Canopy tree	30	516	Wind	9 × 2
	*Sloanea australis*	Elaeocarpaceae	Sahul	CNVF riparian	Canopy tree	45	516	Frugivore	9 × 5
	*Sloanea woollsii*	Elaeocarpaceae	Sahul	CNVF riparian	Canopy tree	45	533.2	Frugivore	9 × 5
	*Syzygium australe*	Myrtaceae	Sahul	NVF riparian	Canopy tree	25	627.8	Frugivore	10 × 20
*Northern‐Local*	*Brachychiton acerifolius*	Malvaceae	Sahul	ANVF—CNVF	Canopy tree	45	361.2	Frugivore	12 × 7
	*Cryptocarya obovata*	Lauraceae	Sahul	CNVF	Canopy tree	45	550.4	Frugivore	10 × 12
	*Diploglottis australis*	Sapindaceae	Sahul	CNVF—SNVF	Canopy persistent secondary	45	597.7	Frugivore	20 × 8
	*Neolitsea dealbata*	Lauraceae	Sunda	CNVF—NVF	Small tree	15	580.5	Frugivore	7 × 9
	*Polyscias murrayi*	Araliaceae	Sunda	CNVF	Mid‐storey pioneer tree	25	344	Frugivore	4 × 2
	*Wilkiea hugeliana*	Monimiaceae	Sahul	Widespread	Small tree	15	571.9	Frugivore	10 × 6

### 2.2 Sequence Data Generation

DNA was extracted from ~100 mg of dried leaf tissue and sequenced by Diversity Arrays Technology (Canberra) using DArTseq (Jaccoud et al. 2001; Kilian et al. 2012), generating thousands of single nucleotide polymorphisms (SNPs) per species. Data filtering was conducted using the *R* packages *dartR* v2.9.7 (Gruber et al. 2018) and *RRtools* (in‐house developed, https://github.com/jasongbragg/RRtools/) in *R* v4.3.1 and RStudio v2024.12.0 + 467 (R Core Team 2023), as part of the Restore and Renew analytical pipeline. Depending on the species‐specific dataset, filtering removed: (1) individuals with > 80%–90% missing loci; (2) sites with < 4–5 individuals; (3) loci with > 20%–30% missing data or with locus reproducibility score < 0.96; (4) excess individuals (> 5 per site, via random subsampling); and (5) SNPs in linkage disequilibrium (one per locus retained). Full details are provided in Supporting Information S1.

### 2.3 Spatial Linearisation for Genetic Analyses

To quantify genetic patterns along a single, ecologically meaningful spatial gradient, we linearised sampling coordinates along the dominant geographic trend of the study system, using a similar approach previously applied in clinal genetic analyses (e.g., Peña et al. 2022). This step was required to support downstream genogeographic analyses, which assume that spatial position can be represented along a single continuous gradient. However, the study rainforests are not distributed along strict latitudinal or longitudinal axes, instead following a broadly linear north–south trend that tracks within ~100 km of the coastline and associated climatic gradients. Using raw latitude as the spatial variable would therefore misrepresent true along‐landscape distances between sites, particularly where the coastline deviates from a single north–south orientation.

To address this, we estimated a best‐fit linear trend through all sampling locations and used this trend as the primary spatial axis for analysis. Specifically, we fitted a linear regression of latitude on longitude across all sites (using *lm* in R; R Core Team 2023) to describe the dominant orientation of the sampling distribution. The slope of this regression was converted to a rotation angle (θ) using the arctangent function, which defines the orientation of a two‐dimensional rotation matrix used to realign site coordinates with the main geographic trend of the rainforest landscape. While beyond the scope of the present study, this framework could be extended to systems where the dominant spatial trend is non‐linear (e.g., rainforest distribution of the entire eastern coastline of Australia) by estimating non‐linear smoothed spatial trajectories (e.g., using loess‐based fits) and projecting sampling locations onto these curves. Additionally, this approach could also be extended to factor elevation by representing spatial structure in higher‐dimensional space, rather than restricting analyses to two‐dimensional geographic coordinates.

Prior to rotation, coordinates were expressed relative to a fixed geographic reference point, defined as the sampled site with the maximum fitted latitude along the regression line (using *predict* from the *stats* R package; R Core Team 2023). This ensured that distances calculated after rotation represented meaningful along‐landscape separation between sites, rather than distances from an arbitrary coordinate origin (Figure 1C). Applying the rotation yielded two orthogonal spatial variables for each site: (1) distance along the fitted geographic axis, and (2) perpendicular distance away from that axis. The rotated axis distance (“rotated latitude”) was used in all downstream analyses comparing genetic variation along the derived latitudinal transect, hereafter defined as the metric *RLatitude*.

### 2.4 Macrogenetic Analyses for Grouping Species

Although this is not a global dataset (as per original definition; Leigh et al. 2021), we use ‘macrogenetic’ to define the scope of this study, considering its considerable scale and the universal applicability of the approaches developed. We applied a series of analyses described below to identify both broad‐scale patterns and fine‐scale groupings across species. These analyses, along with the metrics used, are summarised in Table S2.1.

### 2.5 Net Migration Directionality

To assess net migration direction, we quantified the latitudinal orientation of pairwise migration estimates. Directional migration rates were estimated using the *DivMigrate* function (Sundqvist et al. 2016) in the *diveRsity* v1.9.90 R package (Keenan et al. 2013), applying Jost's D (*D*) as a measure of relative migration strength. For each site pair, the difference in rotated latitude (*ΔRLatitude* = *RLatitude_to*—*RLatitude_from*) was calculated and weighted by the corresponding *DivMigrate* estimate (*ΔRLatitude* × *D*). The mean weighted value for each species captured both the overall direction (northward vs. southward) and intensity of migration. Uncertainty was estimated with a nonparametric bootstrap (1000 replicates; *boot* v1.3–28.1; Canty and Ripley 2022) to generate 95% confidence intervals (CI). Species were considered to exhibit significant net migration directionality if their 95% CI did not overlap zero.

### 2.6 Genogeographic Clustering

To evaluate whether patterns of diversity and migration are consistent across the landscape and form into ecologically meaningful species groups, we applied a modified genogeographic clustering approach across two differing metrics. Originally developed for intertidal species distributed along a closed coastal loop (Arranz et al. 2022), this hierarchical clustering method was adapted here for a linear, terrestrial system characterised by strong latitudinal partitioning. Because absolute values may not be directly comparable across species due to differences in DArTseq marker sets, metrics were normalised within species prior to clustering to allow meaningful comparison of spatial patterns among species.

#### 2.6.1 Broad‐Scale Patterns

For each species, we modelled diversity versus *RLatitude* using a Generalised Additive Model (*gam* function; *mgcv* v.1.9‐0; Wood 2011) with high smoothing (sp = 2) and limited flexibility (knots = 8) to emphasise broad spatial trends. Diversity was measured as normalised expected heterozygosity (*He*/max(*He*) within each species), with *He* estimated using *basicStats* (*diveRsity* v1.9.90; Keenan et al. 2013). Hierarchical clustering of fitted curves was performed with 1000 bootstrap replicates using the *hclust* function (*pvclust* v2.2; Suzuki et al. 2019), with Ward's D2 linkage and Euclidean distance. Clustered groupings were visualised as a dendrogram and support evaluated via AU (approximately unbiased) and BP (bootstrap probability) values, with significant clusters defined as AU ≥ 95% using the *pvrect* function.

#### 2.6.2 Finer‐Scale Groupings

Because species fall into repeated, landscape‐level patterns, we therefore further assessed whether finer‐scale patterns were nested within these major patterns. For species representing each broad‐scale pattern, spatial variation in migration strength was examined using GAM‐smoothed curves of *D* versus |Δ*RLatitude*| (method = ‘REML’, knots = 8) with reduced smoothing to capture finer‐scale gradients. To enable cross‐species comparison of curve shape and trend, each fitted curve was normalised to a common grid of 100 evenly spaced distance values (from 0 to the maximum |Δ*RLatitude*|) using the *approx* function (rule = 2; *stats* package; R Core Team 2023). The same hierarchical clustering and bootstrap resampling procedure was then applied to identify significantly supported groupings within each broader pattern.

### 2.7 Isolation‐By‐Distance

We assessed isolation‐by‐distance (IBD) using pairwise *F*_*ST*_, calculated using *snpgdsFst* (*SNPRelate*; Zheng et al. 2012), and great‐circle distances, calculated using *distHaversine* (geosphere v1.5–18; Hijmans 2022). Species‐specific datasets were grouped according to either broad‐ or fine‐scale patterns identified previously, and average IBD strength per group was evaluated with a linear model. For each regression, the slope, intercept, and coefficient of determination (*R* ^2^) were extracted using *broom* v1.0.5 (Robinson et al. 2023). To test for significant differences in IBD strength, we fitted linear models with interaction terms between geographic distance and species group and extracted estimated slopes of the *F*_*ST*_–distance relationship. Pairwise contrasts of slopes were obtained using estimated marginal trends using *emtrends* (*emmeans* v1.11.1; Lenth 2025).

### 2.8 Climate Data and Analyses

Climatic suitability was modelled for each species across three time periods to assess the influence of climatic fluctuations on predicted habitat availability within the identified species groups, following the approaches of Das et al. (2019) and Dimon et al. (2026). The periods examined were the Last Glacial Maximum (LGM), the present (1989–2014) and the year 2090 under two scenarios: SSP245 (moderate mitigation) and SSP585 (high emissions; Di Virgilio et al. 2022). Ecological niche models (ENMs) were fitted using MaxEnt (Elith et al. 2011; Phillips and Dudík 2008), via the *maxnet* v0.1.4 *R* package (Phillips et al. 2017). Occurrence data spanning each species' full Australian range were obtained from the Atlas of Living Australia (ALA; https://www.ala.org.au) using the *galah* v2.0.0 *R* package (Westgate et al. 2024), with filtering procedures detailed in Supporting Information S3. Seventeen bioclimatic variables were used, excluding Bio08 and Bio09 due to spatial discontinuities arising from instability in the wettest and driest quarter definitions across space, following the same approach as outlined in Dimon et al. (2026). Model performance and additional methodological details are provided in Supporting Information S3.

Binarised suitability maps were generated using the Equal Sensitivity and Specificity (ESS) threshold (Pearson et al. 2006; Table S3.2) to delineate areas of highly probable climatic suitability. To evaluate shared suitability across species, maps were stacked by their respective fine‐scale group (Das et al. 2019) to quantify and visualise the percentage overlap of suitable climates across binned categories. Although analyses focused on the NSW study area, projections were also extended to eastern Australia to place these patterns in a broader spatial context (Figure S3.2).

### 2.9 Regional Genetic Diversity and Turnover

Patterns of regional genetic turnover and diversity were assessed for each group using outputs from Analysis of Molecular Variance (AMOVA) and averaged genetic diversity (*He*) following Dimon et al. (2026). Between‐region AMOVA was calculated with *poppr* v2.9.4 (Kamvar et al. 2014, 2015), and site‐level *He* calculated as previously described. Regions were defined by two major river systems, the Clarence and Hunter Rivers, which represent biogeographic barriers identified in prior studies (e.g., Dimon et al. 2026; Heslewood et al. 2014; Mellick et al. 2013; Milner et al. 2012).

Adjacent regions on either side of each river were compared pairwise, using equalised sample sizes to minimise bias. For each region pair, 100 replicates were run, randomly subsampling sites to match the smaller group. AMOVA results were summarised as the proportion of total genetic variance explained by regional differences, with *p*‐values obtained via permutation tests. Species were grouped according to the identified clusters, and AMOVA variance components were min‐max normalised per species to allow comparison across species and regions (as similar to the genogeographic clustering analyses). Normalised values were then averaged within each species group to produce summary metrics of regional turnover. *He* was calculated per site, min‐max normalised per species, and averaged hierarchically: first across sites within each region, then across species within each identified group.

## 3 Results

### 3.1 Uniformity or Randomness in Landscape Dynamics?

**FIGURE 2:**
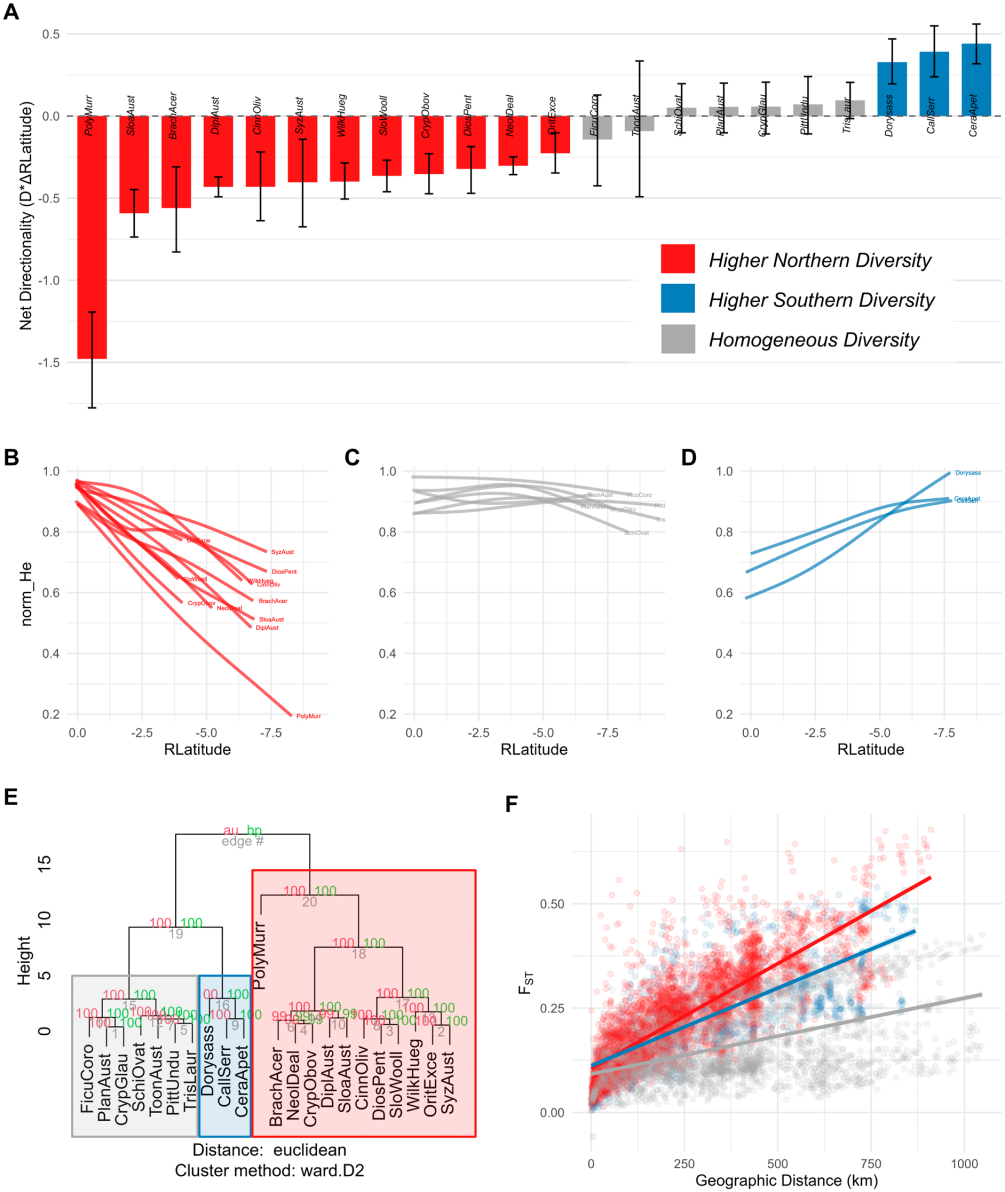
Broad patterns of landscape‐level genetic connectivity and diversity across 22 rainforest species: *Higher Northern Diversity* (red), *Homogeneous Diversity* (grey), and *Higher Southern Diversity* (blue). (A) Species representing each broad pattern are identified by significant directionality in net migration, shown as a two‐way barplot of mean weighted migration (D*Δ*RLatitude*). Negative values indicate net southward migration, while positive values indicate net northward migration. Error bars show 95% bootstrapped confidence intervals, with net migration considered significantly directional if confidence intervals do not overlap zero. (B–D) Genogeographic clustering of bootstrapped, smoothed, normalised *He* versus *RLatitude* curves, with significant clusters corresponding to the same set of species identified through the migration analyses. Significance is indicated by Approximately Unbiased (AU; left) and Bootstrap Probability (BP; right) values in the dendrogram (E). (F) Isolation‐by‐distance (IBD) trends averaged across species representing each identified pattern.

Three broad landscape‐level genetic patterns emerged among the 22 species differing predominantly in significant directionality in net migration (D* ΔRLatitude; Figure 2A), further supported by genogeographic clustering (Figure 2B–E; Figure S4.1A) and averaged IBD slopes (Figure 2F; Figure S4.1C). Twelve species displayed higher diversity in the north with significant north‐to‐south migration (*Higher Northern Diversity* pattern), three species showed the opposite trend (*Higher Southern Diversity* pattern), and the remaining species exhibited more geographically uniform dynamics (*Homogeneous Diversity* pattern). The dendrogram based on genogeographic clustering of smoothed bootstrapped *He* curves revealed significant clustering corresponding to these three patterns, with node support values of AU = 100 for all groups. Additionally, species with *Higher Northern Diversity* and *Higher Southern Diversity* patterns exhibited strong IBD relationships on average (*R* ^2^ = 0.684 and 0.531, respectively), whereas species of the *Homogeneous Diversity* pattern showed a more gradual IBD slope (*R* ^2^ = 0.268).

### 3.2 Replicated Landscape Histories

**FIGURE 3:**
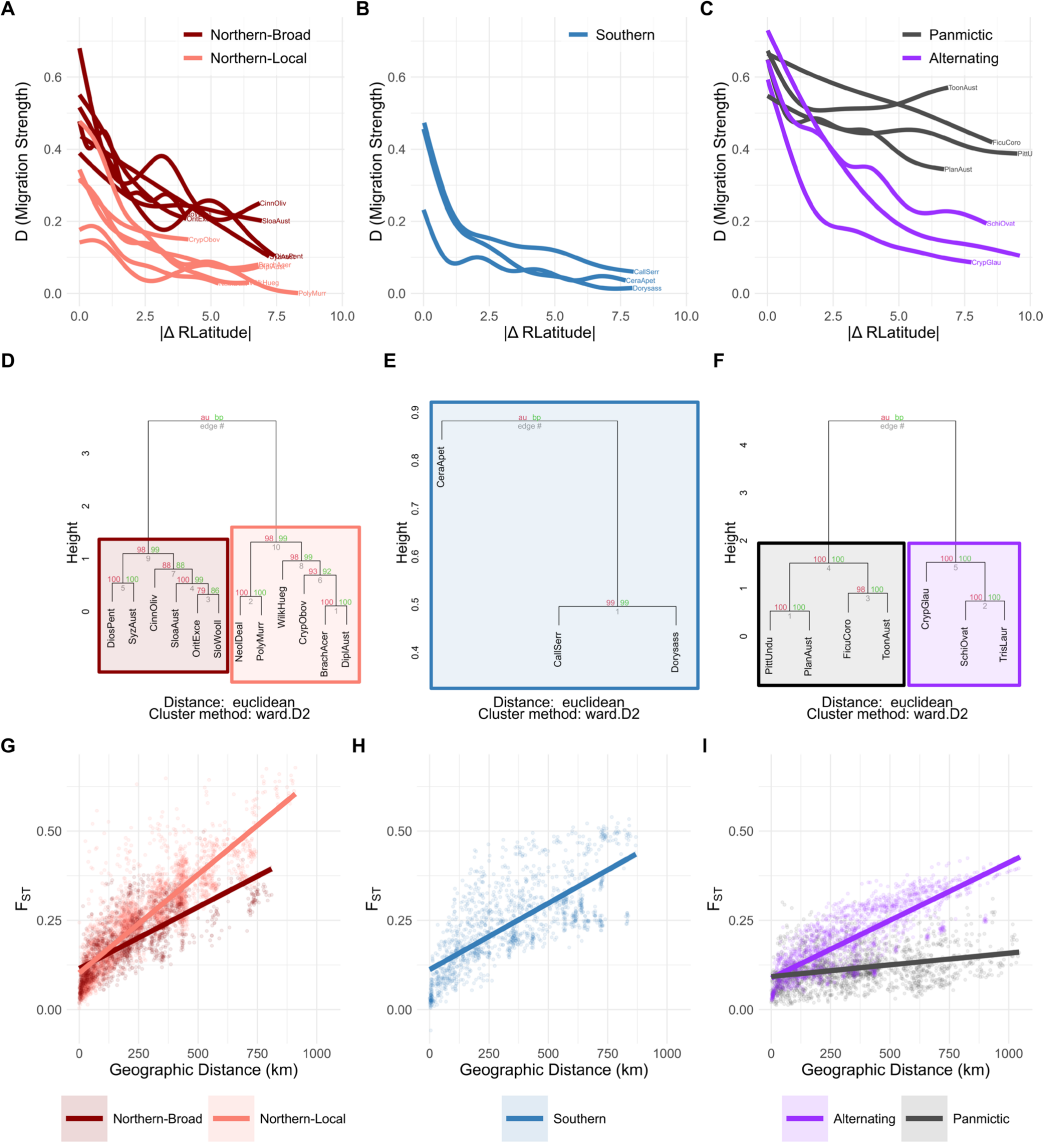
Finer scale species groupings identified through further examination of the broad patterns identified (Left: *Higher Northern Diversity*; Middle: *Higher Southern Diversity*; Right: *Homogeneous Diversity*). Differences among the five identified groups are illustrated through genogeographic clustering of pairwise migration strength (Jost's D; *D*) and the absolute difference in rotated latitude between sites (|Δ*RLatitude*|), visualised as smoothed bootstrapped curves (A–C) normalised to a common grid of 100 evenly spaced distance values (from 0 to the maximum |Δ*RLatitude*|). Dendrograms (D–F) of significant genogeographic clustering are provided for each pattern, with node support given as Approximately Unbiased (AU; left) and Bootstrap Probability (BP; right). Scatterplots with linear regression lines (G–I) visualise significant isolation‐by‐distance (IBD) trends between the identified groups.

Fine‐scale geographic clustering was detected among species representing the three broad‐scale patterns (Figure 3A–F; Figure S4.2A,B). Five distinct species groups emerged from the genogeographic clustering of smoothed migration strength curves (*D* vs. |Δ*RLatitude*|), reflecting differences in how species migrate across the landscape. These groupings displayed significant differences in IBD slopes (Figure 3G–I; Figure S4.2E).

Species within the *Higher Northern Diversity* pattern separated into two significant clusters (AU = 98 for both), each comprising six species (Figure 3A,D; Table 1). Species identified as the *Northern‐Broad* group displayed stronger southward migration than those in the *Northern‐Local* group (Figure 3A). The weaker IBD slopes observed in the *Northern‐Broad* group relative to the *Northern‐Local* group (*R* ^2^ = 0.531 vs. 0.748 respectively; Figure 3G) also reflected greater landscape connectivity for species belonging to the *Northern‐Broad* group.

Significant clustering was also detected among the three species showing the *Higher Southern Diversity* pattern (Figure 3B). However, due to the small sample size, similarity of migration curves and shared northward migration trends (Figure 2A; Figure 3B,H), no further subdivision was applied and these species remained collectively defined as the *Southern* group.

Species displaying the *Homogeneous Diversity* pattern separated into two significant clusters (both AU = 100; Figure 3F). One cluster of four species, termed *Panmictic*, exhibited flatter migration slopes and maintained higher D values across greater distances than the *Alternating* group of three species (Figure 3C). The *Panmictic* group also showed much weaker IBD slopes (*R* ^2^ = 0.099) compared with the *Alternating* group (*R* ^2^ = 0.678), consistent with greater overall landscape connectivity (Figure 3I). In comparison, the *Alternating* group exhibited stronger migration decay and IBD, with irregular distribution of diversity that is less geographically predictable (i.e., areas of high and low diversity alternating across the landscape).

### 3.3 Validating Replicated Landscape Histories by Comparing Strength of Biogeographical Barriers and Species Distribution Modelling

**FIGURE 4:**
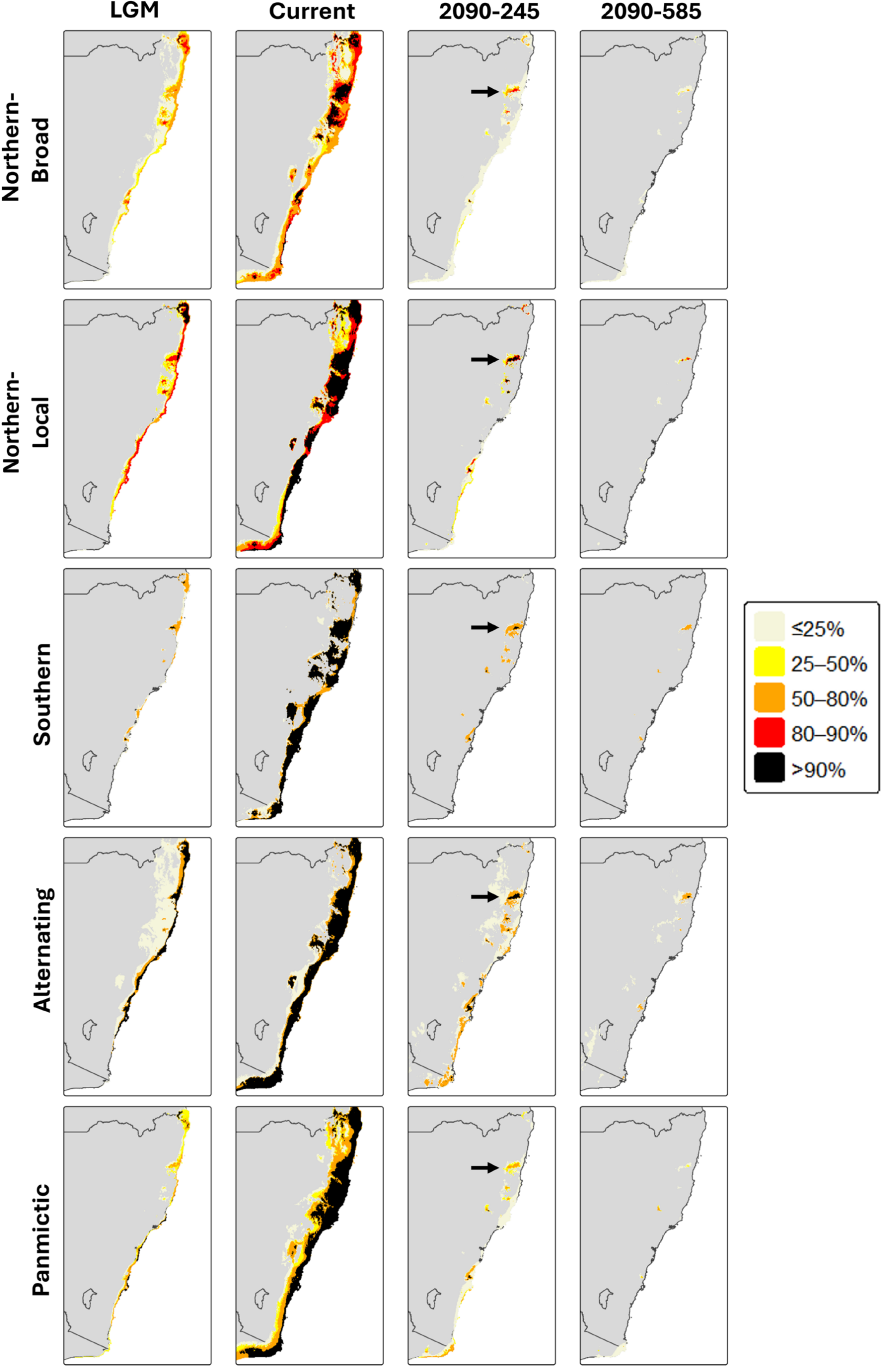
Stacked climatic suitability across past, present, and future conditions for each species group. The past is represented by the Last Glacial Maximum (circa 21 ka, labelled as LGM), the present by the climate from 1989 to 2014 (labelled as Current), and the future by projections for 2090 under two emissions scenarios (SSP245 and SSP585, shown as 2090–245 and 2090–585). In each map, colours represent the percentage of species with suitable climate conditions (above the Equal Sensitivity and Specificity threshold) stacked. The arrow in the plot of 2090–245 indicates the location of Dorrigo where consistently suitable habitat was detected across different groups.

Although the availability of suitable habitat was predicted to consistently expand from the LGM to the present and contract in the future for all species (Figure 4), the five groups displayed distinguishable patterns. The *Northern‐Local* group showed more continuous suitability, both inland and along the coast, than the *Northern‐Broad* group throughout the modelled periods, while the *Southern* group was historically largely restricted to multiple locations across NSW during the LGM. Both the *Panmictic* and *Alternating* groups were modelled with suitable habitat spanning the NSW east coast in the present, but the *Alternating* group retained greater suitability under LGM conditions compared to the *Panmictic* group. Projections under future climate scenarios suggest that suitable habitat will become highly restricted, with the Dorrigo region (and to a lesser extent the Budderoo Plateau/southern Illawarra escarpment) consistently emerging as a suitable future refuge (Figure 4).

**FIGURE 5:**
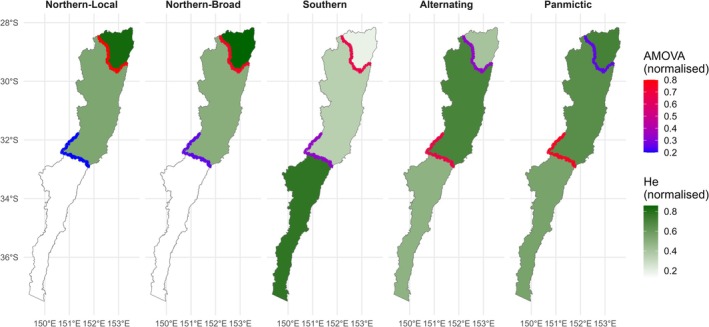
Regional genetic diversity and turnover across the five species groupings identified in this study. The regions studied were based on Dimon et al. (2026), named Northern, Central and Southern, separated by the Clarence River Corridor (CRC, top) and Hunter River Corridor (HRC, bottom), indicated as lines. Line colour, ranging from red (high) to blue (low), represents the normalised between‐group AMOVA variance averaged across species for each grouping. The average normalised expected heterozygosity (*He*) is shown for each region for each group, with the shade of green indicating the level of genetic diversity.

Regional genetic turnover and diversity were assessed across the five species groups based on AMOVA and *He* outputs respectively (Figure 5; Table S4.1,2). Though cross‐species variation in AMOVA values was detected, this was due to some species displaying relatively lower variances (proportion of total genetic variation explained by regional divisions) than others, and general trends of genetic turnover could still be captured by averaging normalised values across species.

On average, *Northern‐Broad*, *Northern‐Local*, and *Southern* groups exhibited the strongest genetic turnover across the Clarence River Corridor (CRC; 0.80 ± 0.45, 0.75 ± 0.50, 0.67 ± 0.58 respectively). While both *Northern‐Broad* and *Northern‐Local* showed higher *He* in the northern regions, the *Southern* group exhibited the greatest *He* in southern regions, which also corresponds to a higher average genetic turnover across the Hunter River Corridor (HRC) when compared to the Northern groups (0.33 ± 0.58 compared to 0.25 ± 0.50 and 0.2 ± 0.45 for *Northern‐Broad* and *Northern‐Local* respectively). On average, both *Panmictic* and *Alternating* groups showed greater turnover across the HRC (0.75 ± 0.5 and 0.67 ± 0.58 respectively), but differed slightly in patterns of diversity, with a more uniform distribution of *He* across all regions, compared to a higher *He* observed in the central region (between the HRC and CRC) for the *Alternating* group.

## 4 Discussion

At broad geographical scales, vegetation assembly patterns reflect both biogeographic history and contemporary ecological events that shape habitat availability and ecosystem function (Ricklefs 2004). At local scales, assembly processes are regulated by species' relative competitiveness within a context of environmental heterogeneity (Van Dyke et al. 2022; Wiens and Donoghue 2004). To interpret assembly‐level processes using Australia's sub‐tropical rainforests as a study system, we applied an innovative, statistically supported, replicable macrogenetic approach across the overlapping distributions of 22 woody species to identify repeated dynamic patterns reflecting shared responses to landscape‐wide filtering processes and with the potential to inform vegetation‐wide management strategies.

### 4.1 Uniformity, Randomness or Replicated Patterns?

The temporal distributional fluctuations of the Australian rainforest vegetation were originally highlighted by palaeoecological evidence, which detected marked distributional contractions during the cool dry glacial peaks, followed by vegetational re‐expansions as conditions improved (Kershaw et al. 2007). Such cyclical adjustments in continental rainforest cover were corroborated by environmental niche modelling studies (Das et al. 2019; Mellick et al. 2012) and phylogeographic evidence, which detected replicated species‐level responses that could be linked to functional properties (Rossetto et al. 2015; Worth et al. 2017; Yap et al. 2020), ecological preferences (Fahey et al. 2019; Rossetto et al. 2009) or shared biogeographic origins (Yap et al. 2022).

Despite similarities in distributions and abundances, the 22 rainforest trees investigated showed neither overall uniformity nor uniquely individual responses to temporal and spatial fluctuations in habitat availability. Instead, three major landscape‐level patterns of genetic diversity emerged (Figure 2). Twelve species displayed higher diversity in the north with a predominant north‐to‐south migration (*Higher Northern Diversity* pattern). Three species displayed the opposite trend (*Higher Southern Diversity* pattern). The remaining species displayed more geographically uniform dynamics (*Homogeneous Diversity* pattern).

Our findings highlight that while previously identified geographic and temporal factors regulate gene flow and shape the distribution of diversity across most study species (e.g., the impact of local biogeographic barriers such as the Hunter River Corridor and the Clarence River Corridor on connectivity; Figure 5), their relative influence varies among groups of species. The identification of distinct but replicated patterns support theoretical expectations that local selective filters can shape species‐level responses to vegetation dynamics and local assembly in distinct ways, even among lineages with shared biogeographic histories or comparable functional attributes (Munoz et al. 2023).

As we established that species groups have different dynamic responses to temporal changes in selective filtering processes, we also confirmed that current overlaps in species distributions and assembly patterns do not necessarily persist as stable ‘vegetational units’ across space and time (Fahey et al. 2024; Willis and Niklas 2004). This is an important consideration when planning long‐term strategies to address ongoing environmental disturbances, as an improved understanding of shared vulnerabilities can lead to increasingly efficacious conservation outcomes (Scoble and Lowe 2010). By characterising replicated dynamic patterns, our macrogenetic approach facilitated the post hoc identification of a suite of functional, ecological and biogeographic features shaping group‐specific distributions and assembly processes.

### 4.2 Multiple Shared Factors Influence Distributional and Assembly Patterns of Emerging Genetic Groups in Our Study System

Species characterised by the *Homogeneous Diversity* pattern display landscape dynamics matching those of species that have recently expanded uniformly across their distribution area (e.g., van der Merwe et al. 2014; Wilde et al. 2021; Yap et al. 2022). Within this pattern, two distinct groups differing in strength of migration emerged (Figure 3). The *Panmictic* group consisting of Sunda‐derived lineages with a relatively recent history on the Australian continent (Miocene to Pleistocene; Crayn et al. 2015; Yap et al. 2018), and mostly represented by early successional, easily dispersed species (Kooyman et al. 2010). The considerably reduced modelled habitat availability during the LGM supports a recent southerly expansion scenario for these Sunda‐derived lineages as habitat suitability improved into the present (Figure 4). In contrast, the *Alternating* group displays a wide extent of modelled LGM refugial areas along the coast, suggesting that contemporary genetic homogeneity likely arose through expansion and admixture from multiple, uniformly distributed refugial sources. This scenario aligns with the longer‐lived Sahul‐derived species that dominate this group and reflects their deeper history on the Australian landscape (Kooyman et al. 2014).

The remaining species show variable levels of gene flow across the study area, with regional concentrations of genetic diversity on either side of two major biogeographic barriers—the Hunter River Corridor and the Clarence River Corridor (Figure 5). The *Higher Northern Diversity* pattern is characterised by common tree species of mixed biogeographic origins that exhibit greater genetic turnover across the Clarence River Corridor in the north and higher genetic diversity in the northern regions (Figure 5). Most of these species are bird dispersed (except for *Orites excelsus* R.Br.) and more common in complex notophyll vine forests (CNVF; Table 1). The *Northern‐Local* group includes smaller trees and pioneers, functionally akin to the *Panmictic* group but with a prevalence of refugial persistence centred around the northern parts of the study area (Figure 4). The *Northern‐Broad* group comprises canopy trees with prolonged local biogeographic histories, mostly of Sahul origins, akin to the *Alternating* group but with most contemporary modelled habitat centred in the north and along the coast of the study area (Figure 4). Although some of the metrics are similar between these two groups as they both comprise species with long‐term persistence in the study area, species within the *Alternating* group persisted across multiple, latitudinally heterogeneous refugia rather than localised northern ones.

Finally, the three species in the *Southern* group display higher genetic turnover across the HRC in the south and higher genetic diversity within the southern regions (Figure 5). These species are of Sahul origins and are more common in simple notophyll vine forests (SNVF; Table 1), with *Ceratopetalum apetalum* D.Don displaying strong latitudinal structure typical of a species with a long‐standing presence on the landscape. Interestingly modelled LGM refugia for this group were not confined to southern areas (Figure 4), suggesting that assembly dynamics are more complex than would be expected under simplified ‘warm versus cool’ rainforest types.

The objective of this study was not to explicitly explore the phylogeography of Australian subtropical rainforests. While the groupings identified from this study system may need slight adjustments as additional species are analysed, they offer a pragmatic advancement in understanding assembly patterns and phylogeographic processes, as well as directly informing biodiversity management. This is because regional strategies developed for groups of species with similar landscape‐level histories can provide improved interpretative and predictive frameworks for guiding community‐wide management (Zbinden et al. 2023), even in complex circumstances such as those associated with climate change (Verboom et al. 2010).

Within such context, our findings suggest that restoration (or other active management) strategies for the highly dynamic *Homogeneous Diversity* species may require relatively little guidance and local interpretative knowledge. Genetic diversity is evenly distributed across their ranges, dispersal‐mediated gene flow is extensive, and modelled future habitat availability is high (in fact the highest for the *Alternating* group) and broadly uniform (Figure 4). In contrast, active management of all other species will require more careful consideration as genomic diversity is more regionally concentrated, and future refugia (sensu Rossetto and Kooyman 2021) are more scattered and restricted (Figure 4). From a landscape‐level management perspective, it is worth noting the modelled importance of the Dorrigo Plateau (Northern and New England Tablelands of NSW) as an area of climatic stability in future climate scenarios, somewhat unexpectedly for *Southern* group species, demonstrating how strategies based on perceived correlations between latitudinal location and habitat persistence can be overly simplistic.

### 4.3 Conclusion

Our study demonstrates how standardised comparative landscape‐genomic approaches can identify groupings that reflect shared histories and assembly processes, providing an interpretational framework to inform improved management strategies. We also demonstrate how interpretative generalisations derived from pre‐defined functional or ecological characteristics (or from a limited number of species) are unlikely to be as informative or predictive. By empirically quantifying and comparing genetic diversity relative to spatial and environmental distributions (i.e., measuring outcomes), it is possible to identify species that have converged on similar distributional dynamics, even when a priori expectations based on specific traits would suggest otherwise. Applying these methods to larger numbers of co‐occurring species across diverse vegetation types, promises to reveal the multiple interacting factors that shape community assembly processes across spatial and temporal scales.

In an applied multispecies context, the identification of replicated dynamic patterns instigated by temporal changes in selective pressures and their subsequent interpretation within the setting of contemporary assembly patterns is a valuable contribution to the development of improved biodiversity management procedures. It is also important to note that attaining macrogenetic‐based outcomes is no longer a limitation: the necessary data need not be exhaustive, can be collected quickly and cost‐effectively, can be regularly updated and can be used to answer a wide range of additional theoretical and applied questions.

## Author Contributions

Maurizio Rossetto conceived the idea for this study with input from all co‐authors. Jia‐Yee S. Yap and Richard Dimon assembled and analysed the data. Maurizio Rossetto led manuscript writing with all co‐authors contributing to revisions.

## Funding

This research was supported by the Foundation and Friends of the Botanic Gardens of Sydney and by the NSW Environmental Trust through the *Science Saving Rainforests* project. Relevant Environmental Trust grants were awarded to the Big Scrub Rainforest Conservancy (2019/RD/0006; 2019/RR/0098) and the Big Scrub Foundation (2020/RR/0105).

## Conflicts of Interest

The authors declare no conflicts of interest.

## Supporting information


**Supporting Information: S1.** Supplementary methods for genomic data filtering, screening, and dataset characteristics.
**Supporting Information: S2.** Summary of statistical methods and diversity metrics applied to broad‐scale and fine‐scale groupings.
**Supporting Information: S3.** Supplementary methods and outputs relating to environmental niche modelling.
**Supporting Information: S4.** Additional outputs from Landscape‐level analyses.
**Table S1.1.** Species‐specific dataset characteristics and filtering parameters. *Locus missingness* indicates the maximum allowed proportion of missingness per SNP prior to filtering. *Filtered SNPs* is the total number of retained SNPs within each dataset. *Min N per site* is the minimum threshold used for inclusion of a sampling site; *Avg N per site* is the average number of individuals per site after filtering (*N Samples/N Sites*).
**Table S2.1.** A summary of the questions, statistical analyses and diversity metrics applied to each analysis in this study to identify and compare both broad‐scale patterns and fine‐scale groupings. *RLatitude* corresponds to latitude of each population after a rotation matrix centered around a reference point is applied. *ΔRLatitude* therefore represents the difference in rotated latitude between pairwise population comparisons (*ΔRLatitude* = *R*Latitude_to − *R*Latitude_from). *D* refers to Jost's‐D metric obtained through *DivMigrate* analyses of pairwise population comparisons. FST refers to pairwise fixation index values calculated through *SNPrelate*. *He* refers to expected heterozygosity calculated through *diveRsity*. Analysis of Molecular Variance (AMOVA) was calculated through *poppr*. Species occurrence data was obtained through the Atlas of Living Australia (ALA).
**Table S3.1.** Model performance assessed using the area under the receiver operating characteristic curve (AUC) and maximum True Skill Statistic (maxTSS). Standard deviation (SD) values for each measure provided.
**Table S3.2.** Equal Sensitivity and Specificity (ESS) threshold for each species‐specific model.
**Figure S3.1.** Area (km^2^) of modelled habitat suitability based on ESS threshold for the LGM, the present and 2090 for the emission scenarios SSP245 and SSP585 for each species of each group.
**Figure S3.2.** Stacked climatic suitability across past, present, and future conditions for each group across the entire east coast of Australia. The past is represented by the Last Glacial Maximum (~21 ka, labelled as LGM), the present by the climate from 1989 to 2014 (labelled as Current), and the future by projections for 2090 under two emissions scenarios (SSP245 and SSP585, shown as 2090–245 and 2090–585). In each map, colours represent the percentage of species within each group with suitable climate conditions, based on cells exceeding the Equal Sensitivity and Specificity threshold.
**Figure S4.1.** Additional outputs corresponding to the patterns shown in Figure 2 of the main text. (A) Principal components analysis (PC1 vs. PC2) of hierarchical clustering (*pvclust*) based on normalised *He* versus *RLatitude* genogeographic curves, with species coloured by their assigned broad‐scale pattern. (B) Barplots showing average population pairwise FST for each species, coloured by their respective broad‐scale pattern. (C) Forest plots showing significant isolation‐by‐distance (IBD) slopes with 95% confidence intervals for all species within each pattern group. (D) Forest plots of IBD slopes for each individual species.
**Figure S4.2.** Additional outputs corresponding to the patterns shown in Figure 3 of the main text. (A, B) Principal components analysis (PC1 vs. PC2) of hierarchical clustering (*pvclust*) based on normalised *D* versus |Δ*RLatitude*| genogeographic curves, with species coloured by their assigned fine‐scale species grouping (Higher Southern Diversity pattern excluded due to limited sample size for clustering). Barplots showing average population pairwise *F*_*ST*_, averaged across species for each grouping (C) and for each individual species (D), coloured by their respective fine‐scale species grouping. Forest plots showing significant isolation‐by‐distance (IBD) slopes with 95% confidence intervals for all species within each group (E) and for individual species (F).
**Table S4.1.** Species‐level Analysis of Molecular Variance (AMOVA) results across the Clarence River Corridor (CRC; Northern vs. Central regions) and Hunter River Corridor (HRC: Central vs. Southern regions) barriers. For each species, the table reports the genetic variation (Sigma) partitioned between and within samples from neighbouring regions (between all populations within the designated region north and south of each barrier), and the corresponding *p*‐values testing the significance of among‐region differentiation. These raw values underpin the scaled and normalised summaries shown in Figure 5 and Table S4.2. Average variation is calculated after 100 repetitions, with each test based on 999 permutations (*p*‐value) through the *randtest* function from the R Package, *ade4* v 1.7–22 (Dray & Dufour 2007).
**Table S4.2.** Group‐level summary of AMOVA results. Values represent the mean (± SD) of the normalised between‐region variance for each of the five species groups across the Clarence River Corridor (CRC; Northern vs. Central regions) and Hunter River Corridor (HRC: Central vs. Southern regions) barriers, as used to colour the lines in Figure 5.

## Data Availability

Data available through Dryad: DOI: 10.5061/dryad.fxpnvx131.
