# Supplementary material for: Macrogenetic Alignment in Ecological Strategies Better Interprets Assembly Processes Than Pre‐Determined Functional Groupings
Source: Ecol Lett. 2026 Jun 7;29(6):e70418. doi: 10.1111/ele.70418 (PMC13243724; doi:10.1111/ele.70418)
Supplement: Supplementary file 1 — Supporting Information: S1. Supplementary methods for genomic data filtering, screening, and dataset characteristics. Supporting Information: S2. Summary of statistical methods and diversity metrics applied to broad‐scale and fine‐scale groupings. Supporting Information: S3. Supplementary methods and outputs relating to environmental niche modelling. Supporting Information: S4. Additional outputs from Landscape‐level analyses. Table S1.1. Species‐specific dataset characteristics and filtering parameters. Locus missingness indicates the maximum allowed proportion of missingness per SNP prior to filtering. Filtered SNPs is the total number of retained SNPs within each dataset. Min N per site is the minimum threshold used for inclusion of a sampling site; Avg N per site is the average number of individuals per site after filtering (N Samples/N Sites). Table S2.1. A summary of the questions, statistical analyses and diversity metrics applied to each analysis in this study to identify and compare both broad‐scale patterns and fine‐scale groupings. RLatitude corresponds to latitude of each population after a rotation matrix centered around a reference point is applied. ΔRLatitude therefore represents the difference in rotated latitude between pairwise population comparisons (ΔRLatitude = RLatitude_to − RLatitude_from). D refers to Jost's‐D metric obtained through DivMigrate analyses of pairwise population comparisons. FST refers to pairwise fixation index values calculated through SNPrelate. He refers to expected heterozygosity calculated through diveRsity. Analysis of Molecular Variance (AMOVA) was calculated through poppr. Species occurrence data was obtained through the Atlas of Living Australia (ALA). Table S3.1. Model performance assessed using the area under the receiver operating characteristic curve (AUC) and maximum True Skill Statistic (maxTSS). Standard deviation (SD) values for each measure provided. Table S3.2. Equal Sensitivity and Specificity (ESS) threshol [file ELE-29-0-s001.docx]

**Supporting Information – “Macrogenetic alignment in ecological strategies better interprets assembly processes than pre-determined functional groupings”**

**Supporting information S1: Supplementary methods for genomic data filtering, screening, and dataset characteristics**

*Initial SNP filtering and quality control*

To minimise genotyping error and ensure comparability across taxa, the following sequential filters were applied to each species dataset:

1. Sample-level filtering: exclude any sample with >80% missing loci .
2. Site-level filtering: exclude sites with fewer than 4-5 samples. The minimum threshold (4 or 5) varied among species and is reported in the column *Min N per site* in **Table S1.1**.
3. Locus-level filtering: SNPs were retained only if (i) the proportion of missingness was 20–30% or less (species-specific thresholds are given in the column *Locus missingness* in **Table S1.1**), (ii) reproducibility scores were ≥0.96. Where more than a single SNP was present per sequenced locus, subsampling was performed to retain a single SNP per locus to avoid linkage bias.
4. Sample subsampling: Where more than 5 samples were genotyped from a site, subsampling to five samples was implemented to standardise within-site representation. This led to most sites being represented by five individuals, with some sites represented by four (**Table S1.1**).

In addition, samples showing evidence of misidentification, hybridisation, duplication, or cultivation were removed prior to downstream analysis. This was performed through a series of preliminary analyses as outlined below.

*Detection of potential clonality and relatedness*

To detect clonality (e.g., from vegetative reproduction), pairwise kinship coefficient was calculated between samples within each population. This calculation applied the PLINK method via the *snpgdsIBDMoM* function in the SNPRelate v.1.33.1 package (Zheng *et al.* 2012), involving the SNP data being filtered with a minor allele frequency (MAF) threshold of ≥0.05. Samples were considered genetically identical when the pairwise kinship coefficient exceeded 0.354 (Manichaikul *et al.* 2010). In such cases, a single, random representative of each genet is retained. Sites were excluded from downstream analyses if this filtering reduced the number of samples below the species-specific minimum threshold.

*Exploratory population structure screening*

Preliminary analyses were conducted on the filtered SNP datasets to detect genetic outliers within each species. While this study focused on natural populations in more remote areas, such outliers can (in other cases) result from planted individuals at an unknowingly sampled restoration site, species field misidentification, or potential hybridisation with nearby species. These analyses included:

1. Principal components analysis (PCA) using adegenet v2.1.0 (Jombart 2008) to visualise multivariate patterns of genetic variation.
2. Splitstree network analysis using a genetic distance matrix with RSplitsTree v0.1.0 (Bickel & Zakharko 2016) and tanggle v1.8.0 (Schliep *et al.* 2023) to explore network topologies and possible introgression.
3. Ancestral group assignment using the *sNMF* function in *LEA* v3.10.2 (Frichot & François 2015) for K = 2-5 to identify potential ancestral groupings and admixture.

Concordance among PCA, network analyses, and LEA outputs was used to identify and remove any potential genetic outliers from each species dataset.

**Table S1.1:** Species-specific dataset characteristics and filtering parameters. *Locus missingness* indicates the maximum allowed proportion of missingness per SNP prior to filtering. *Filtered SNPs* is the total number of retained SNPs within each dataset. *Min N per site* is the minimum threshold used for inclusion of a sampling site; *Avg N per site* is the average number of individuals per site after filtering (*N Samples/N Sites*).

| species | Species Short | Locus Missingness | Sample missingness | Filtered SNPS | N Samples | N Sites | Min N per Site | Avg N per Site |
| --- | --- | --- | --- | --- | --- | --- | --- | --- |
| *Brachychiton acerifolius* | BrachAcer | 0.2 | 0.8 | 13533 | 100 | 20 | 5 | 5 |
| *Callicoma serratifolia* | CallSerr | 0.2 | 0.8 | 602 | 145 | 29 | 5 | 5 |
| *Ceratopetalum apetalum* | CeraApet | 0.2 | 0.8 | 558 | 192 | 39 | 4 | 4.92 |
| *Cinnamomum oliveri* | CinnOliv | 0.2 | 0.8 | 10381 | 105 | 21 | 5 | 5 |
| *Cryptocarya glaucescens* | CrypGlau | 0.2 | 0.8 | 28375 | 140 | 28 | 5 | 5 |
| *Cryptocarya obovata* | CrypObov | 0.2 | 0.8 | 14174 | 110 | 22 | 5 | 5 |
| *Diospyros pentamera* | DiosPent | 0.2 | 0.8 | 12453 | 150 | 30 | 5 | 5 |
| *Diploglottis australis* | DiplAust | 0.2 | 0.8 | 7520 | 275 | 55 | 5 | 5 |
| *Doryphora sassafras* | Dorysass | 0.2 | 0.8 | 2218 | 135 | 27 | 5 | 5 |
| *Ficus coronata* | FicuCoro | 0.2 | 0.8 | 12800 | 130 | 26 | 5 | 5 |
| *Neolitsea dealbata* | NeolDeal | 0.2 | 0.8 | 6758 | 290 | 58 | 5 | 5 |
| *Orites excelsus* | OritExce | 0.2 | 0.8 | 14652 | 115 | 23 | 5 | 5 |
| *Pittosporum undulatum* | PittUndu | 0.2 | 0.8 | 11465 | 230 | 46 | 5 | 5 |
| *Planchonella australis* | PlanAust | 0.2 | 0.8 | 23518 | 160 | 32 | 5 | 5 |
| *Polyscias murrayi* | PolyMurr | 0.2 | 0.8 | 5549 | 120 | 24 | 5 | 5 |
| *Schizomeria ovata* | SchiOvat | 0.2 | 0.8 | 13418 | 160 | 32 | 5 | 5 |
| *Sloanea australis* | SloaAust | 0.2 | 0.8 | 6648 | 135 | 27 | 5 | 5 |
| *Sloanea woollsii* | SloWooll | 0.2 | 0.8 | 11458 | 150 | 30 | 5 | 5 |
| *Syzygium australe* | SyzAust | 0.3 | 0.9 | 481 | 80 | 17 | 4 | 4.71 |
| *Toona ciliata* | ToonCili | 0.2 | 0.8 | 17718 | 85 | 18 | 4 | 4.72 |
| *Tristaniopsis laurina* | TrisLaur | 0.2 | 0.8 | 19151 | 220 | 44 | 5 | 5 |
| *Wilkiea hugeliana* | WilkHueg | 0.2 | 0.8 | 20056 | 140 | 28 | 5 | 5 |

**Supporting information S2: Summary of statistical methods and diversity metrics applied to broad-scale and fine-scale groupings**

**Table S2.1:** A summary of the questions, statistical analyses and diversity metrics applied to each analysis in this study to identify and compare both broad-scale patterns and fine-scale groupings. ***RLatitude*** corresponds to latitude of each population after a rotation matrix centered around a reference point is applied. ***ΔRLatitude*** therefore represents the difference in rotated latitude between pairwise population comparisons (*ΔRLatitude* = *R*Latitude_to − *R*Latitude_from). ***D*** refers to Jost’s-D metric obtained through *DivMigrate* analyses of pairwise population comparisons. FST refers to pairwise fixation index values calculated through *SNPrelate*. ***He*** refers to expected heterozygosity calculated through *diveRsity*. Analysis of Molecular Variance (**AMOVA**) was calculated through *poppr*. Species occurrence data was obtained through the Atlas of Living Australia (**ALA**)

| Scale | Question | Analysis / Method | Metric / Variable |
| --- | --- | --- | --- |
| Broad-scale patterns | Is there a significant net directionality in migration across species? | Nonparametric bootstrap | ***ΔRLatitude*********D*** |
|  | Do species cluster by broad scale genogeographic patterns? | Hierarchical clustering | GAM-smoothed normalised ***He*** vs. ***R*Latitude** |
|  | Do grouped IBD trends differ in slope among species patterns? | Pairwise contrasts of estimated marginal trends | Linear smoothing of pairwise **FST** vs. great-circle distance |
| Fine-scale groupings | Do grouped IBD trends differ in slope among fine-scale species groups? | Pairwise contrasts of estimated marginal trends | Linear smoothing of pairwise **FST** vs. great-circle distance |
|  | Do species groups cluster by fine scale genogeographic patterns? | Hierarchical clustering | GAM-smoothed normalised ***D*** vs. **\|Δ*R*Latitude\|** |
|  | How do suitable habitats between species groups shift across time periods? | Ecological niche modelling (historic, current, future) | Species occurrence data (**ALA**) |
|  | How does regional genetic diversity vary spatially? | Visual comparison | Average normalised ***He*** |
|  | How does genetic turnover vary among regions? | Visual comparison | Average normalised between-group **AMOVA** variance |

**Supporting information S3: Supplementary methods and outputs relating to environmental niche modelling**

*Climate data*

The occurrence data that was applied spans each species’ full distribution in Australia, obtained from the Atlas of Living Australia (ALA; <https://www.ala.org.au>) using the *galah* v2.0.0 R package (Westgate *et al.* 2024). Records were cleaned with the *processALA* v0.5.5 R package and by manual inspection to remove cultivated individuals and spatial outliers arising from misidentification or incorrect GPS coordinates.

Climate data representing baseline or “current” conditions (1989 to 2014) was downloaded from the ANUClimate data repository (<https://dapds00.nci.org.au/thredds/catalogs/gh70/catalog.html>) at 0.01° (~1 km) resolution. Historic LGM and future climate projections were sourced from the Climate Modelling Inter-comparison Project 6 (CMIP6) repository of models generated for the IPCC 6th Assessment Report (<https://pcmdi.llnl.gov/CMIP6/>). Two scenarios for future climate change in 2090 (Shared Socioeconomic Pathways, SSPs) were used: SSP245 (moderate mitigation) and SSP585 (high emissions). We used all available LGM data (4 climate models) and the selection of climate models for future climate based on the recommendations of Di Virgilio *et al.* (2022).

ENMs were fitted using the MaxEnt modelling method (Elith *et al.* 2011; Phillips & Dudík 2008), implemented through the *maxnet* v0.1.4 R package (Phillips *et al.* 2017). MaxEnt models output a suitability score for each grid cell ranging from 0 to 1, with a score of 1 representing a grid cell’s perfect match to optimal climate. Model performance was assessed using area under the receiver operating characteristic curve (AUC; Swets, 1988) and true skill statistic, TSS (**Table S3.1**).

Although the focus of our study was within NSW, we also display the outputs across the entire mainland of Australia here (Fig. S3.2).

**Table S3.1:** Model performance assessed using the area under the receiver operating characteristic curve (AUC) and maximum True Skill Statistic (maxTSS). Standard deviation (SD) values for each measure provided.

| Species | AUC | SD (AUC) | maxTSS | SD (maxTSS) |
| --- | --- | --- | --- | --- |
| *Brachychiton acerifolius* | 0.8859 | 0.0102 | 0.6403 | 0.0193 |
| *Callicoma serratifolia* | 0.9348 | 0.004 | 0.7439 | 0.012 |
| *Ceratopetalum apetalum* | 0.9303 | 0.0053 | 0.735 | 0.0143 |
| *Cinnamomum oliveri* | 0.9317 | 0.0089 | 0.7164 | 0.024 |
| *Cryptocarya glaucescens* | 0.9323 | 0.0032 | 0.7411 | 0.011 |
| *Cryptocarya obovata* | 0.8946 | 0.0071 | 0.628 | 0.019 |
| *Diospyros pentamera* | 0.9163 | 0.0069 | 0.7173 | 0.0133 |
| *Diploglottis australis* | 0.912 | 0.0055 | 0.6993 | 0.0112 |
| *Doryphora sassafras* | 0.9161 | 0.0053 | 0.6833 | 0.0141 |
| *Ficus coronata* | 0.9094 | 0.0067 | 0.6928 | 0.015 |
| *Neolitsea dealbata* | 0.9202 | 0.0045 | 0.7292 | 0.0098 |
| *Orites excelsus* | 0.9301 | 0.0078 | 0.7514 | 0.0267 |
| *Pittosporum undulatum* | 0.8976 | 0.0025 | 0.6726 | 0.0098 |
| *Planchonella australis* | 0.904 | 0.0063 | 0.681 | 0.0219 |
| *Polyscias murrayi* | 0.9233 | 0.0069 | 0.7154 | 0.0193 |
| *Schizomeria ovata* | 0.9203 | 0.004 | 0.7186 | 0.014 |
| *Sloanea australis* | 0.931 | 0.0068 | 0.7278 | 0.0218 |
| *Sloanea woollsii* | 0.9276 | 0.0054 | 0.7315 | 0.0233 |
| *Syzygium australe* | 0.9026 | 0.0041 | 0.6827 | 0.0159 |
| *Toona ciliata* | 0.9157 | 0.0065 | 0.7006 | 0.0189 |
| *Tristaniopsis laurina* | 0.9114 | 0.0035 | 0.7041 | 0.0109 |
| *Wilkiea hugeliana* | 0.9212 | 0.003 | 0.7025 | 0.0102 |

**
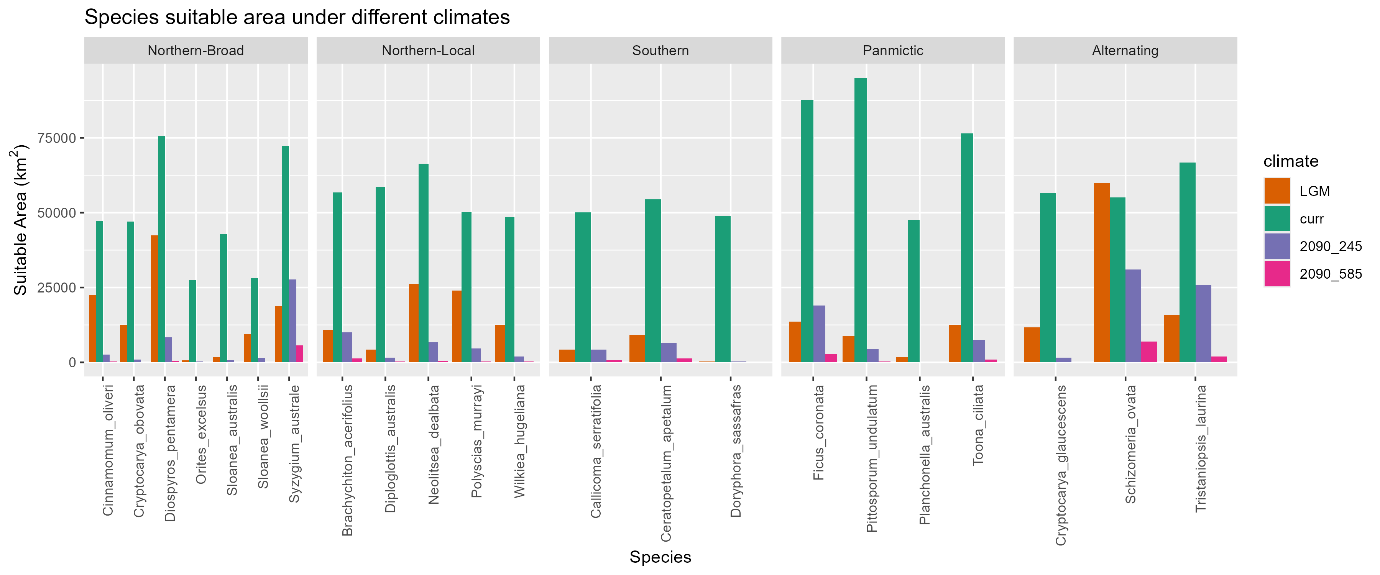
**

**Figure S3.1**: Area (km^2^) of modelled habitat suitability based on ESS threshold for the LGM, the present and 2090 for the emission scenarios SSP245 and SSP585 for each species of each group.

**Table S3.2**: Equal Sensitivity and Specificity (ESS) threshold for each species-specific model.

| Species | ESS |
| --- | --- |
| *Brachychiton acerifolius* | 0.44 |
| *Callicoma serratifolia* | 0.4 |
| *Ceratopetalum apetalum* | 0.41 |
| *Cinnamomum oliveri* | 0.37 |
| *Cryptocarya glaucescens* | 0.4 |
| *Cryptocarya obovata* | 0.41 |
| *Diospyros pentamera* | 0.42 |
| *Diploglottis australis* | 0.46 |
| *Doryphora sassafras* | 0.43 |
| *Ficus coronata* | 0.42 |
| *Neolitsea dealbata* | 0.42 |
| *Orites excelsus* | 0.33 |
| *Pittosporum undulatum* | 0.48 |
| *Planchonella australis* | 0.41 |
| *Polyscias murrayi* | 0.35 |
| *Schizomeria ovata* | 0.42 |
| *Sloanea australis* | 0.38 |
| *Sloanea woollsii* | 0.36 |
| *Toona ciliata* | 0.38 |
| *Syzygium australe* | 0.42 |
| *Tristaniopsis laurina* | 0.4 |
| *Wilkiea hugeliana* | 0.35 |


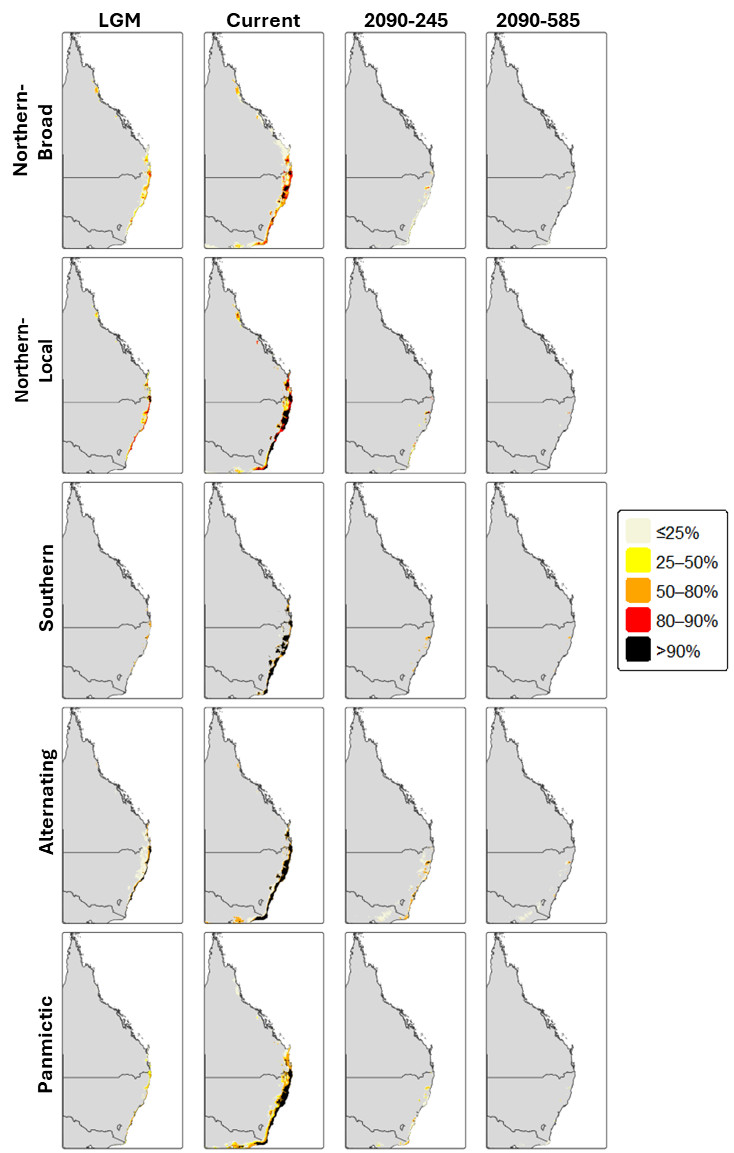


**Figure S3.2**: Stacked climatic suitability across past, present, and future conditions for each group across the entire east coast of Australia. The past is represented by the Last Glacial Maximum (~21 ka, labelled as LGM), the present by the climate from 1989 to 2014 (labelled as Current), and the future by projections for 2090 under two emissions scenarios (SSP245 and SSP585, shown as 2090-245 and 2090-585). In each map, colours represent the percentage of species within each group with suitable climate conditions, based on cells exceeding the Equal Sensitivity and Specificity threshold.

**Supporting information S4: Additional outputs from Landscape-level analyses**

For the “Identifying broad-scale pattern” section, a further clustering of the bootstrapped curves involved visualising the output as a Principal Components Analysis (PCA) using the *prcomp* function (R Core Team 2023) as shown below.


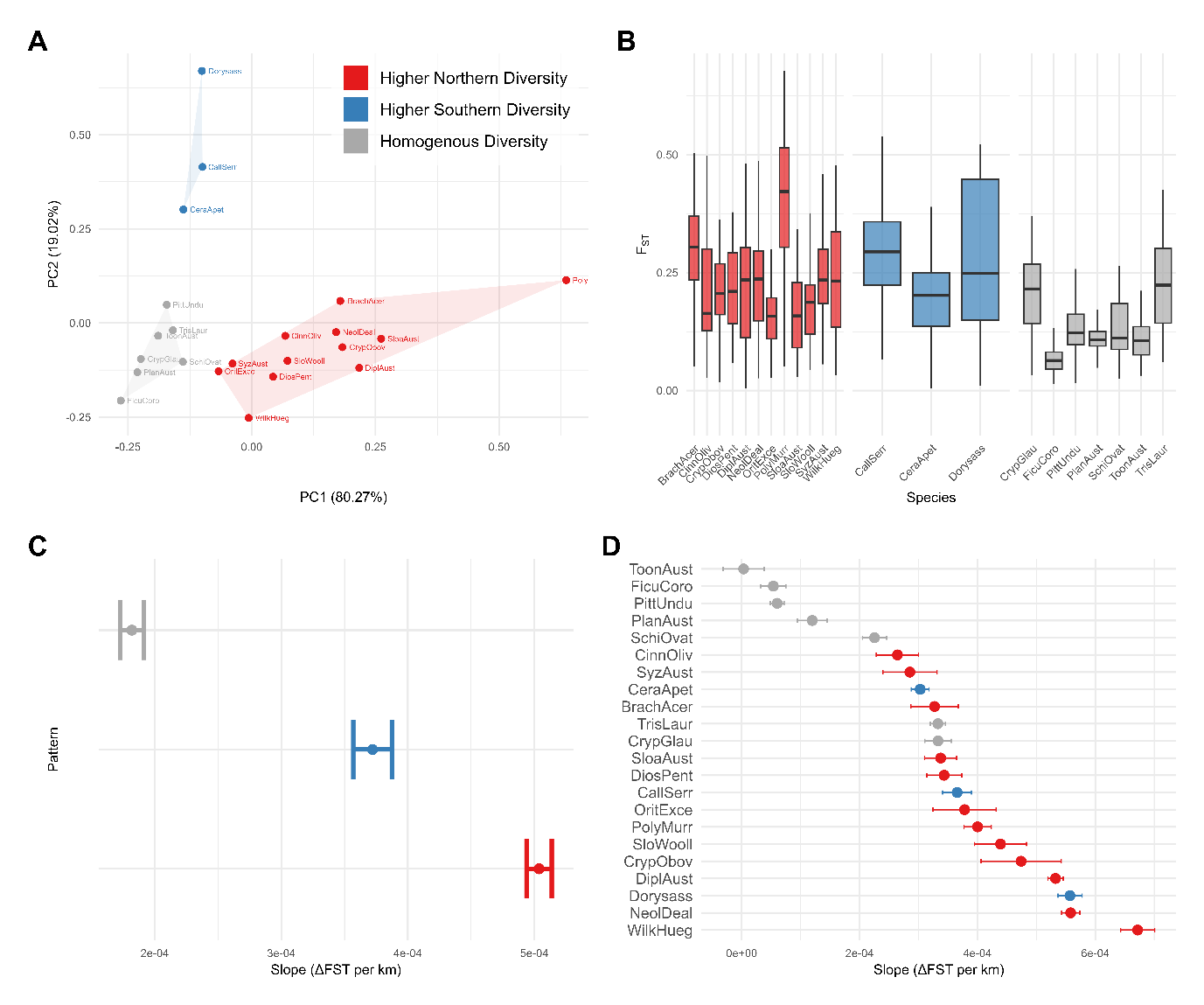


**Figure S4.1:** Additional outputs corresponding to the patterns shown in **Fig. 2** of the main text. **(A)** Principal components analysis (PC1 vs. PC2) of hierarchical clustering (*pvclust*) based on normalised *He* vs *RLatitude* genogeographic curves, with species coloured by their assigned broad-scale pattern. **(B)** Barplots showing average population pairwise FST for each species, coloured by their respective broad-scale pattern. **(C)** Forest plots showing significant isolation-by-distance (IBD) slopes with 95% confidence intervals for all species within each pattern group. **(D)** Forest plots of IBD slopes for each individual species.


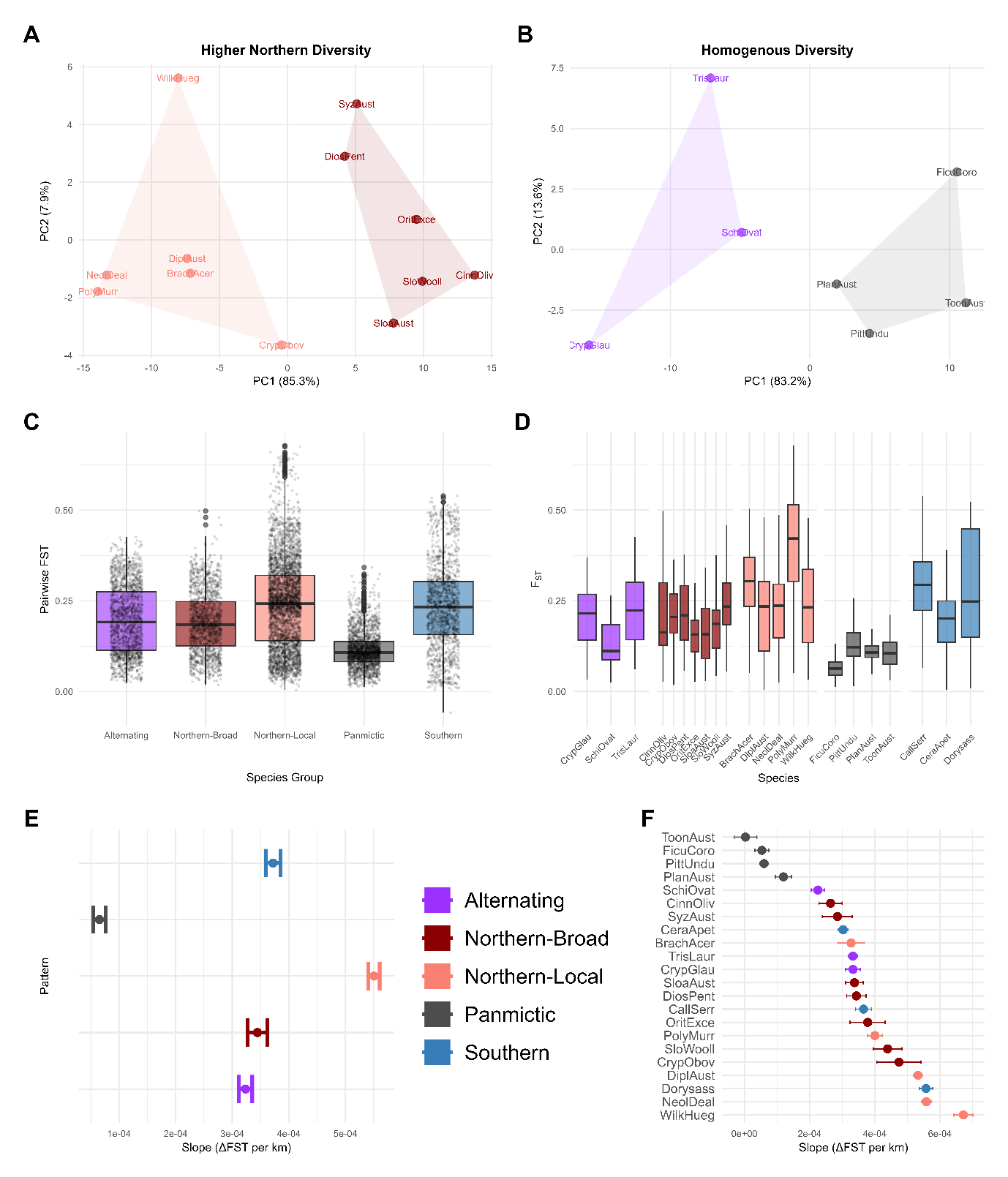


**Figure S4.2:** Additional outputs corresponding to the patterns shown in **Fig. 3** of the main text. (**A-B**) Principal components analysis (PC1 vs. PC2) of hierarchical clustering (*pvclust*) based on normalised *D* vs |Δ*RLatitude*| genogeographic curves, with species coloured by their assigned fine-scale species grouping (Higher Southern Diversity pattern excluded due to limited sample size for clustering). Barplots showing average population pairwise *F_ST_*, averaged across species for each grouping (**C**) and for each individual species (**D**), coloured by their respective fine-scale species grouping. Forest plots showing significant isolation-by-distance (IBD) slopes with 95% confidence intervals for all species within each group (**E**) and for individual species (**F**).

**Table S4.1**: Species-level Analysis of Molecular Variance (AMOVA) results across the Clarence River Corridor (CRC; Northern vs Central regions) and Hunter River Corridor (HRC: Central vs Southern regions) barriers. For each species, the table reports the genetic variation (Sigma) partitioned between and within samples from neighbouring regions (between all populations within the designated region north and south of each barrier), and the corresponding P-values testing the significance of among-region differentiation. These raw values underpin the scaled and normalised summaries shown in **Fig. 5** and **Table S4.2**. Average variation is calculated after 100 repetitions, with each test based on 999 permutations (pvalue) through the *randtest* function from the R Package, *ade4* v 1.7-22 (Dray & Dufour 2007).

| Source | Sigma | pvalue | n_reps | Region1 | Region2 | Species | No. equal samples per region |
| --- | --- | --- | --- | --- | --- | --- | --- |
| Total variations | 548.9903437 | 0.01 | 100 | Central | Northern | BrachAcer | 30 |
| Variations Between samples | 89.14819167 | 0.01 | 100 | Central | Northern | BrachAcer | 30 |
| Variations Within samples | 459.8421521 | 0.01 | 100 | Central | Northern | BrachAcer | 30 |
| Total variations | 584.8932212 | 0.01 | 100 | Central | Southern | BrachAcer | 20 |
| Variations Between samples | 165.7983061 | 0.01 | 100 | Central | Southern | BrachAcer | 20 |
| Variations Within samples | 419.0949151 | 0.01 | 100 | Central | Southern | BrachAcer | 20 |
| Total variations | 39.0643 | 0.01 | 100 | Central | Northern | CallSerr | 10 |
| Variations Between samples | 13.44727222 | 0.01 | 100 | Central | Northern | CallSerr | 10 |
| Variations Within samples | 25.61702778 | 0.01 | 100 | Central | Northern | CallSerr | 10 |
| Total variations | 35.17970978 | 0.01 | 100 | Central | Southern | CallSerr | 65 |
| Variations Between samples | 7.502918083 | 0.01 | 100 | Central | Southern | CallSerr | 65 |
| Variations Within samples | 27.67679169 | 0.01 | 100 | Central | Southern | CallSerr | 65 |
| Total variations | 30.06496886 | 0.01 | 100 | Central | Northern | CeraApet | 54 |
| Variations Between samples | 5.805171459 | 0.01 | 100 | Central | Northern | CeraApet | 54 |
| Variations Within samples | 24.25979741 | 0.01 | 100 | Central | Northern | CeraApet | 54 |
| Total variations | 28.22439074 | 0.01 | 100 | Central | Southern | CeraApet | 54 |
| Variations Between samples | 5.313895412 | 0.01 | 100 | Central | Southern | CeraApet | 54 |
| Variations Within samples | 22.91049533 | 0.01 | 100 | Central | Southern | CeraApet | 54 |
| Total variations | 339.5469071 | 0.01 | 100 | Central | Northern | CinnOliv | 30 |
| Variations Between samples | 38.23879317 | 0.01 | 100 | Central | Northern | CinnOliv | 30 |
| Variations Within samples | 301.308114 | 0.01 | 100 | Central | Northern | CinnOliv | 30 |
| Total variations | 359.6984 | 0.01 | 100 | Central | Southern | CinnOliv | 10 |
| Variations Between samples | 73.16926111 | 0.01 | 100 | Central | Southern | CinnOliv | 10 |
| Variations Within samples | 286.5291389 | 0.01 | 100 | Central | Southern | CinnOliv | 10 |
| Total variations | 1841.296824 | 0.01 | 100 | Central | Northern | CrypGlau | 15 |
| Variations Between samples | 396.4212616 | 0.01 | 100 | Central | Northern | CrypGlau | 15 |
| Variations Within samples | 1444.875562 | 0.01 | 100 | Central | Northern | CrypGlau | 15 |
| Total variations | 1765.133144 | 0.01 | 100 | Central | Southern | CrypGlau | 60 |
| Variations Between samples | 367.6938698 | 0.01 | 100 | Central | Southern | CrypGlau | 60 |
| Variations Within samples | 1397.439274 | 0.01 | 100 | Central | Southern | CrypGlau | 60 |
| Total variations | 606.1564115 | 0.01 | 100 | Central | Northern | CrypObov | 40 |
| Variations Between samples | 90.6410615 | 0.01 | 100 | Central | Northern | CrypObov | 40 |
| Variations Within samples | 515.51535 | 0.01 | 100 | Central | Northern | CrypObov | 40 |
| Total variations | 419.3076451 | 0.01 | 100 | Central | Northern | DiosPent | 55 |
| Variations Between samples | 80.07368248 | 0.01 | 100 | Central | Northern | DiosPent | 55 |
| Variations Within samples | 339.2339626 | 0.01 | 100 | Central | Northern | DiosPent | 55 |
| Total variations | 311.7171 | 0.01 | 100 | Central | Southern | DiosPent | 10 |
| Variations Between samples | 36.40279444 | 0.01 | 100 | Central | Southern | DiosPent | 10 |
| Variations Within samples | 275.3143056 | 0.01 | 100 | Central | Southern | DiosPent | 10 |
| Total variations | 625.5501547 | 0.01 | 100 | Central | Northern | DiplAust | 105 |
| Variations Between samples | 144.9388026 | 0.01 | 100 | Central | Northern | DiplAust | 105 |
| Variations Within samples | 480.611352 | 0.01 | 100 | Central | Northern | DiplAust | 105 |
| Total variations | 513.7595169 | 0.01 | 100 | Central | Southern | DiplAust | 30 |
| Variations Between samples | 82.38025489 | 0.01 | 100 | Central | Southern | DiplAust | 30 |
| Variations Within samples | 431.379262 | 0.01 | 100 | Central | Southern | DiplAust | 30 |
| Total variations | 42.07825824 | 0.01 | 100 | Central | Northern | Dorysass | 40 |
| Variations Between samples | 7.862793826 | 0.01 | 100 | Central | Northern | Dorysass | 40 |
| Variations Within samples | 34.21546441 | 0.01 | 100 | Central | Northern | Dorysass | 40 |
| Total variations | 121.9577385 | 0.01 | 100 | Central | Southern | Dorysass | 40 |
| Variations Between samples | 52.92905506 | 0.01 | 100 | Central | Southern | Dorysass | 40 |
| Variations Within samples | 69.02868347 | 0.01 | 100 | Central | Southern | Dorysass | 40 |
| Total variations | 968.6898091 | 0.01 | 100 | Central | Northern | FicuCoro | 24 |
| Variations Between samples | 29.00600934 | 0.01 | 100 | Central | Northern | FicuCoro | 24 |
| Variations Within samples | 939.6837997 | 0.01 | 100 | Central | Northern | FicuCoro | 24 |
| Total variations | 973.3320145 | 0.01 | 100 | Central | Southern | FicuCoro | 24 |
| Variations Between samples | 33.88606675 | 0.01 | 100 | Central | Southern | FicuCoro | 24 |
| Variations Within samples | 939.4459477 | 0.01 | 100 | Central | Southern | FicuCoro | 24 |
| Total variations | 183.4443688 | 0.01 | 100 | Central | Northern | NeolDeal | 135 |
| Variations Between samples | 51.02505356 | 0.01 | 100 | Central | Northern | NeolDeal | 135 |
| Variations Within samples | 132.4193153 | 0.01 | 100 | Central | Northern | NeolDeal | 135 |
| Total variations | 132.8573278 | 0.01 | 100 | Central | Southern | NeolDeal | 15 |
| Variations Between samples | 24.66640867 | 0.01 | 100 | Central | Southern | NeolDeal | 15 |
| Variations Within samples | 108.1909192 | 0.01 | 100 | Central | Southern | NeolDeal | 15 |
| Total variations | 903.4490342 | 0.01 | 100 | Central | Northern | OritExce | 35 |
| Variations Between samples | 163.4402867 | 0.01 | 100 | Central | Northern | OritExce | 35 |
| Variations Within samples | 740.0087475 | 0.01 | 100 | Central | Northern | OritExce | 35 |
| Total variations | 564.2690779 | 0.01 | 100 | Central | Northern | PittUndu | 30 |
| Variations Between samples | 25.88527271 | 0.01 | 100 | Central | Northern | PittUndu | 30 |
| Variations Within samples | 538.3838052 | 0.01 | 100 | Central | Northern | PittUndu | 30 |
| Total variations | 554.4852311 | 0.01 | 100 | Central | Southern | PittUndu | 75 |
| Variations Between samples | 17.09368603 | 0.01 | 100 | Central | Southern | PittUndu | 75 |
| Variations Within samples | 537.391545 | 0.01 | 100 | Central | Southern | PittUndu | 75 |
| Total variations | 723.3915031 | 0.01 | 100 | Central | Northern | PlanAust | 55 |
| Variations Between samples | 29.84640087 | 0.01 | 100 | Central | Northern | PlanAust | 55 |
| Variations Within samples | 693.5451022 | 0.01 | 100 | Central | Northern | PlanAust | 55 |
| Total variations | 786.489186 | 0.01 | 100 | Central | Southern | PlanAust | 15 |
| Variations Between samples | 33.24214022 | 0.01 | 100 | Central | Southern | PlanAust | 15 |
| Variations Within samples | 753.2470458 | 0.01 | 100 | Central | Southern | PlanAust | 15 |
| Total variations | 1195.123432 | 0.01 | 100 | Central | Northern | PolyMurr | 25 |
| Variations Between samples | 519.4881943 | 0.01 | 100 | Central | Northern | PolyMurr | 25 |
| Variations Within samples | 675.635238 | 0.01 | 100 | Central | Northern | PolyMurr | 25 |
| Total variations | 609.827463 | 0.01 | 100 | Central | Southern | PolyMurr | 45 |
| Variations Between samples | 153.2997633 | 0.01 | 100 | Central | Southern | PolyMurr | 45 |
| Variations Within samples | 456.5276996 | 0.01 | 100 | Central | Southern | PolyMurr | 45 |
| Total variations | 360.9302757 | 0.01 | 100 | Central | Northern | SchiOvat | 55 |
| Variations Between samples | 29.19780572 | 0.01 | 100 | Central | Northern | SchiOvat | 55 |
| Variations Within samples | 331.73247 | 0.01 | 100 | Central | Northern | SchiOvat | 55 |
| Total variations | 615.7000285 | 0.01 | 100 | Central | Southern | SchiOvat | 24 |
| Variations Between samples | 102.7165594 | 0.01 | 100 | Central | Southern | SchiOvat | 24 |
| Variations Within samples | 512.9834691 | 0.01 | 100 | Central | Southern | SchiOvat | 24 |
| Total variations | 249.962666 | 0.01 | 100 | Central | Northern | SloaAust | 25 |
| Variations Between samples | 48.5675486 | 0.01 | 100 | Central | Northern | SloaAust | 25 |
| Variations Within samples | 201.3951174 | 0.01 | 100 | Central | Northern | SloaAust | 25 |
| Total variations | 140.44085 | 0.01 | 100 | Central | Southern | SloaAust | 10 |
| Variations Between samples | 19.76887778 | 0.01 | 100 | Central | Southern | SloaAust | 10 |
| Variations Within samples | 120.6719722 | 0.01 | 100 | Central | Southern | SloaAust | 10 |
| Total variations | 904.6950321 | 0.01 | 100 | Central | Northern | SloWooll | 60 |
| Variations Between samples | 132.9095162 | 0.01 | 100 | Central | Northern | SloWooll | 60 |
| Variations Within samples | 771.7855159 | 0.01 | 100 | Central | Northern | SloWooll | 60 |
| Total variations | 19.9146 | 0.1273 | 100 | Central | Northern | SyzAust | 5 |
| Variations Between samples | 1.56585 | 0.1273 | 100 | Central | Northern | SyzAust | 5 |
| Variations Within samples | 18.34875 | 0.1273 | 100 | Central | Northern | SyzAust | 5 |
| Total variations | 0.810191761 | 0.306704545 | 100 | Central | Southern | SyzAust | 8 |
| Variations Between samples | 0.185800528 | 0.306704545 | 100 | Central | Southern | SyzAust | 8 |
| Variations Within samples | 0.624391234 | 0.306704545 | 100 | Central | Southern | SyzAust | 8 |
| Total variations | 1352.355396 | 0.01 | 100 | Central | Northern | ToonCili | 19 |
| Variations Between samples | 49.21764884 | 0.01 | 100 | Central | Northern | ToonCili | 19 |
| Variations Within samples | 1303.137748 | 0.01 | 100 | Central | Northern | ToonCili | 19 |
| Total variations | 1413.064209 | 0.01 | 100 | Central | Southern | ToonCili | 32 |
| Variations Between samples | 57.11539104 | 0.01 | 100 | Central | Southern | ToonCili | 32 |
| Variations Within samples | 1355.948818 | 0.01 | 100 | Central | Southern | ToonCili | 32 |
| Total variations | 1427.193263 | 0.01 | 100 | Central | Northern | TrisLaur | 25 |
| Variations Between samples | 111.3123807 | 0.01 | 100 | Central | Northern | TrisLaur | 25 |
| Variations Within samples | 1315.880882 | 0.01 | 100 | Central | Northern | TrisLaur | 25 |
| Total variations | 1484.997195 | 0.01 | 100 | Central | Southern | TrisLaur | 85 |
| Variations Between samples | 380.5482761 | 0.01 | 100 | Central | Southern | TrisLaur | 85 |
| Variations Within samples | 1104.448919 | 0.01 | 100 | Central | Southern | TrisLaur | 85 |
| Total variations | 1553.490962 | 0.01 | 100 | Central | Northern | WilkHueg | 30 |
| Variations Between samples | 558.4076432 | 0.01 | 100 | Central | Northern | WilkHueg | 30 |
| Variations Within samples | 995.0833185 | 0.01 | 100 | Central | Northern | WilkHueg | 30 |
| Total variations | 1317.541482 | 0.01 | 100 | Central | Southern | WilkHueg | 40 |
| Variations Between samples | 230.1222864 | 0.01 | 100 | Central | Southern | WilkHueg | 40 |
| Variations Within samples | 1087.419196 | 0.01 | 100 | Central | Southern | WilkHueg | 40 |

**Table S4.2:** Group-level summary of AMOVA results. Values represent the mean (± SD) of the normalised between-region variance for each of the five species groups across the Clarence River Corridor (CRC; Northern Vs Central regions) and Hunter River Corridor (HRC: Central vs Southern regions) barriers, as used to colour the lines in **Fig. 5**.

| Species Group | Region1 | Region2 | Area of Turnover | Mean var norm | Sd var norm | n | Se var norm | Mean pair var |
| --- | --- | --- | --- | --- | --- | --- | --- | --- |
| Alternating | Central | Southern | HRC | 0.667 | 0.577 | 3 | 0.333 | 0.44 |
| Northern-Broad | Central | Southern | HRC | 0.250 | 0.500 | 4 | 0.250 | 0.44 |
| Northern-Local | Central | Southern | HRC | 0.200 | 0.447 | 5 | 0.200 | 0.44 |
| Panmictic | Central | Southern | HRC | 0.750 | 0.500 | 4 | 0.250 | 0.44 |
| Southern | Central | Southern | HRC | 0.333 | 0.577 | 3 | 0.333 | 0.44 |
| Alternating | Central | Northern | CRC | 0.333 | 0.577 | 3 | 0.333 | 0.56 |
| Northern-Broad | Central | Northern | CRC | 0.750 | 0.500 | 6 | 0.204 | 0.56 |
| Northern-Local | Central | Northern | CRC | 0.800 | 0.447 | 6 | 0.183 | 0.56 |
| Panmictic | Central | Northern | CRC | 0.250 | 0.500 | 4 | 0.250 | 0.56 |
| Southern | Central | Northern | CRC | 0.667 | 0.577 | 3 | 0.333 | 0.56 |

**References**

Bickel, B. & Zakharko, T. (2016). *RSplitsTree: SplitsTree file generation and invoking from R*.

Di Virgilio, G., Ji, F., Tam, E., Nishant, N., Evans, J.P., Thomas, C., *et al.* (2022). Selecting CMIP6 GCMs for CORDEX dynamical downscaling: Model performance, independence, and climate change signals. *Earth’s Future*, 10, e2021EF002625.

Dray, S. & Dufour, A.-B. (2007). The ade4 Package: Implementing the Duality Diagram for Ecologists. *Journal of Statistical Software*, 22, 1–20.

Elith, J., Phillips, S.J., Hastie, T., Dudík, M., Chee, Y.E. & Yates, C.J. (2011). A statistical explanation of MaxEnt for ecologists. *Diversity and distributions*, 17, 43–57.

Frichot, E. & François, O. (2015). LEA: An R package for landscape and ecological association studies. *Methods in Ecology and Evolution*, 6, 925–929.

Jombart, T. (2008). adegenet: a R package for the multivariate analysis of genetic markers. *Bioinformatics*, 24, 1403–1405.

Manichaikul, A., Mychaleckyj, J.C., Rich, S.S., Daly, K., Sale, M. & Chen, W.-M. (2010). Robust relationship inference in genome-wide association studies. *Bioinformatics*, 26, 2867–2873.

Phillips, S.J., Anderson, R.P., Dudík, M., Schapire, R.E. & Blair, M.E. (2017). Opening the black box: An open-source release of Maxent. *Ecography*, 40, 887–893.

Phillips, S.J. & Dudík, M. (2008). Modeling of species distributions with Maxent: new extensions and a comprehensive evaluation. *Ecography*, 31, 161–175.

R Core Team. (2023). *R: A Language and Environment for Statistical Computing*. R Foundation for Statistical Computing, Vienna, Austria.

Schliep, K., Vidal-Garcia, M., Solis-Lemus, C., Biancani, L., Ada, E. & Diaz, L.F.H. (2023). *tanggle: Visualization of Phylogenetic Networks*.

Swets, J.A. (1988). Measuring the accuracy of diagnostic systems. *Science*, 240, 1285–1293.

Westgate, M., Stevenson, M., Kellie, D. & Newman, P. (2024). *galah: Biodiversity Data from the GBIF Node Network*.

Zheng, X., Levine, D., Shen, J., Gogarten, S.M., Laurie, C. & Weir, B.S. (2012). A high-performance computing toolset for relatedness and principal component analysis of SNP data. *Bioinformatics*, 28, 3326–3328.
